# Trajectories of urea‑to‑creatinine ratio and risk of clinical outcomes in survivors of acute kidney disease: a population-based study

**DOI:** 10.1093/ckj/sfaf175

**Published:** 2025-05-29

**Authors:** Heng-Chih Pan, Jui-Yi Chen, Nai-Chi Teng, Fang-Yu Yeh, Chun Yin See, Chiao-Yin Sun, Vin-Cent Wu, Likwang Chen

**Affiliations:** Graduate Institute of Clinical Medicine, College of Medicine, National Taiwan University, Taipei, Taiwan; Chang Gung University College of Medicine, Taoyuan, Taiwan; Division of Nephrology, Department of Internal Medicine, Keelung Chang Gung Memorial Hospital, Keelung, Taiwan; Community Medicine Research Center, Keelung Chang Gung Memorial Hospital, Keelung, Taiwan; Kidney Research Center and Department of Nephrology, Linkou Chang Gung Memorial Hospital, Taoyuan, Taiwan; Division of Nephrology, Department of Internal Medicine, Chi Mei Medical Center, Tainan, Taiwan; Department of Health and Nutrition, Chia Nan University of Pharmacy and Science, Tainan, Taiwan; Institute of Population Health Sciences, National Health Research Institutes, Zhunan, Miaoli, Taiwan; Division of Nephrology, Department of Internal Medicine, National Taiwan University Hospital, Taipei, Taiwan; Division of Nephrology, Department of Internal Medicine, National Cheng Kung University Hospital, College of Medicine, National Cheng Kung University, Tainan, Taiwan; Division of Nephrology, Department of Internal Medicine, Keelung Chang Gung Memorial Hospital, Keelung, Taiwan; Division of Nephrology, Department of Internal Medicine, National Taiwan University Hospital, Taipei, Taiwan; NSARF (National Taiwan University Hospital Study Group of ARF), TAIPAI (Taiwan Primary Aldosteronism Investigators), and CAKS (Taiwan Consortium for Acute Kidney Injury and Kidney Diseases), Taipei, Taiwan; Institute of Population Health Sciences, National Health Research Institutes, Zhunan, Miaoli, Taiwan

**Keywords:** acute kidney disease, major adverse kidney events, mortality, trajectory, urea-to-creatinine ratio

## Abstract

**Background:**

The urea-to-creatinine ratio (UCR) serves as a common metric for assessing dehydration, catabolism, excessive protein intake and impaired kidney perfusion. However, the performance of UCR in patients with acute kidney disease (AKD) remains unexplored.

**Methods:**

In this retrospective cohort study, we enrolled 6703 survivors of AKD from a nationwide population-based database in Taiwan linked with laboratory data from 1 January 2015 to 31 December 2018. Using a group-based trajectory model (GBTM), we identified UCR trajectories and investigated their dynamic changes. We associated these trajectories with major adverse kidney events (MAKEs) as the primary outcome, and mortality and major adverse cardiovascular events (MACEs) as secondary outcomes in AKD survivors.

**Results:**

A total of 9717 AKD survivors were enrolled with a mean follow-up of 1.3 ± 0.9 years. The incidence of MAKEs was 43.7%, the incidence of mortality was 26.3% and the incidence of MACEs was 31.1%. After adjusting for known covariates, UCR trajectories independently predicted MAKEs, all-cause mortality and MACEs. Compared with the middle trajectory group, the high UCR trajectory group had a significantly elevated risk of MAKEs [hazard ratio (HR) 1.54, 95% confidence interval (CI) 1.38–1.73], mortality rate (HR 1.59, 95% CI 1.41–1.80) and MACEs (HR 1.57, 95% CI 1.40–1.77). In contrast, the low UCR trajectory group had an increased risk of MAKEs (HR 1.30, 95% CI 1.20–1.41) and a reduced risk of mortality rate (HR 0.84, 95% CI 0.74–0.95).

**Conclusions:**

Distinct UCR trajectories predicted MAKEs, all-cause mortality and MACEs in AKD survivors. A high UCR trajectory was associated with the highest risk of adverse events, whereas a low UCR trajectory carried a higher risk of MAKEs but a lower risk of mortality. These findings underscore the clinical relevance of monitoring UCR trajectories for long-term prognosis and risk stratification in AKD patients.

KEY LEARNING POINTS **What was known:**

- The urea-to-creatinine ratio (UCR) is commonly used to assess dehydration, catabolism and kidney perfusion, but its prognostic value in survivors of acute kidney disease (AKD) had not been fully explored.

**This study adds:**

- Distinct UCR trajectories independently predict major adverse kidney events, all-cause mortality and major adverse cardiovascular events in AKD survivors, offering important insights into their long-term prognosis.

**Potential impact:**

- Monitoring UCR trajectories may enhance risk stratification and long-term management of AKD survivors, potentially guiding more personalized care strategies to improve outcomes.

## INTRODUCTION

The ramifications of acute kidney injury (AKI) can be multifaceted, ranging from chronic kidney disease (CKD) to cardiovascular events, bone fractures, and even mortality [1, 2]. The severity of AKI is closely correlated with an elevated risk of adverse events and mortality, and those experiencing dialysis-requiring AKI (AKI-D), the most severe form, face the bleakest prognosis [3, 4].

The kidneys play a crucial role in filtering blood, and blood urea nitrogen (BUN) and creatinine (Cr) are commonly used to assess kidney function [5]. Both BUN and Cr levels usually rise proportionally as kidney function declines [6, 7]. However, several other factors can also cause an increase in BUN levels, including heart failure, excessive protein intake, gastrointestinal bleeding and catabolism [8]. As a result of neurohormonal activities and kidney function regulatory processes, an increase in BUN may occur disproportionately to serum Cr (sCr), leading to an elevated urea-to-creatinine ratio (UCR). This elevated UCR serves as a biochemical hallmark of persistent critical illness, and could potentially serve as a surrogate for catabolism [9]. Various factors, including dehydration, catabolism and diminished kidney blood flow may contribute to this phenomenon [8, 10].

In addition, elevated UCR levels are associated with adverse cardiac outcomes in AKI patients, such as acute myocardial infarction, ischemic stroke and heart failure [11–14]. However, the connection between dynamic changes in UCR and the long-term impact on the kidneys in patients with acute kidney disease (AKD) remains less understood. To address this knowledge gap, we employed a group-based trajectory model (GBTM) in this study to investigate UCR trajectories and their dynamic changes in survivors of AKD. A GBTM is a semi-parametric method for clustering individuals with similar developmental trajectories of a variable into the most likely latent subgroups based on posterior probabilities [15]. This study identified distinct UCR trajectories over time, and the GBTM provided more holistic insights into the evolution of UCR and its implications for patient health.

## MATERIALS AND METHODS

### Data source, study protocol and patient selection

**Figure 1:**
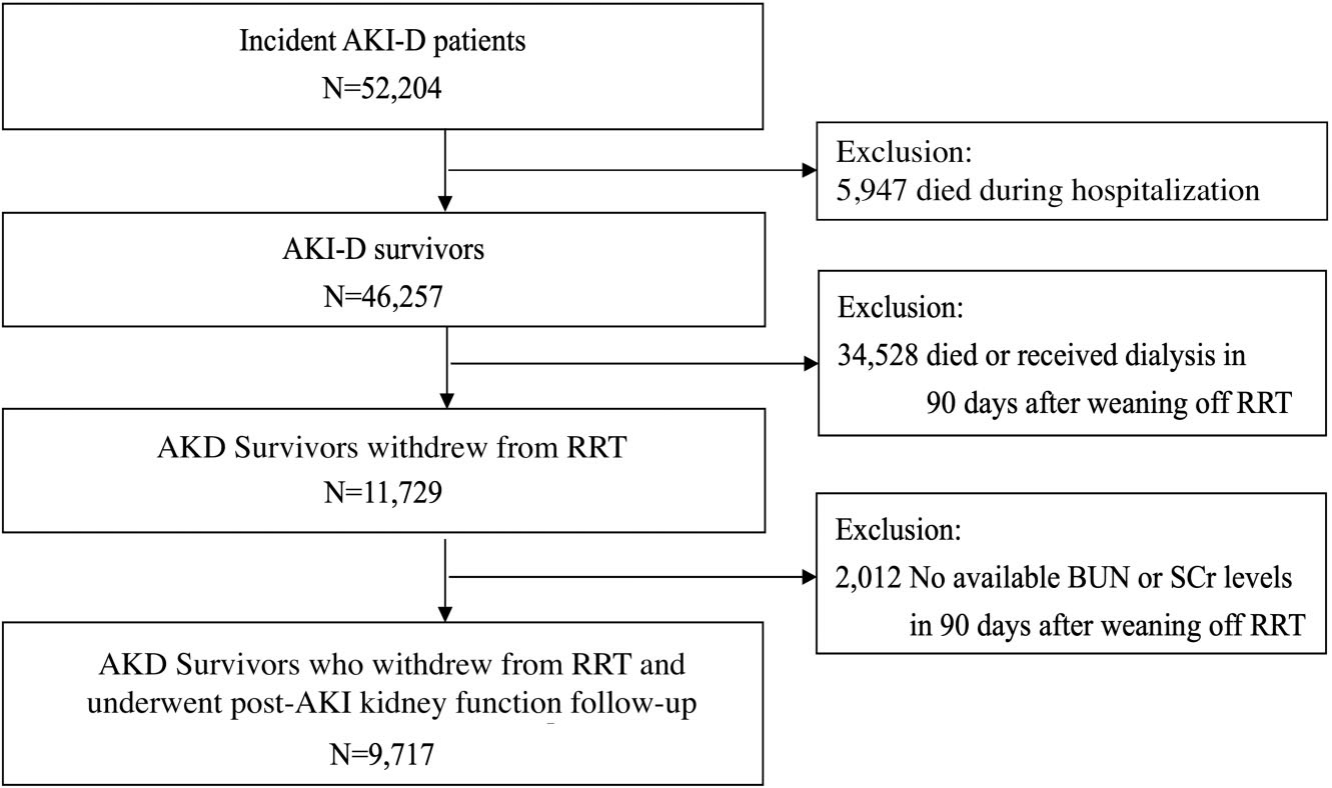
Algorithm depicting the study flowchart. This flowchart outlines the selection process of the study participants. Out of 52 204 incident AKI-D patients, 46 257 AKI-D survivors were identified. After excluding 5947 patients who died during hospitalization and 34 528 patients who either died or received dialysis within 90 days post-RRT weaning, a total of 11 729 AKD survivors remained. A further 2012 patients were excluded due to unavailable BUN or SCr levels within 90 days, resulting in a final cohort of 9717 AKD survivors who underwent post-AKI-D kidney function follow-up.

Data for this study were extracted from the National Health Insurance Research Database (NHIRD), a robust clinical database in Taiwan which has been used in numerous impactful epidemiological investigations [16–19]. Initiated in 1995, the NHIRD, one of the world's largest databases of its kind, encompasses nearly all (99%) inpatient and outpatient claims for the population of Taiwan, exceeding 22 million people (Fig. 1). The National Health Insurance (NHI) Administration conducts regular audits of data and records from healthcare institutions and providers to identify potential insurance fraud, thereby ensuring the accuracy of the NHIRD [16, 17, 20, 21]. In addition to the standard claims-based data, we accessed an extended dataset from the Applied Health Research Data Integration Service, which the NHI Administration has offered since 2015. This platform integrates additional laboratory parameters such as blood urea nitrogen, serum creatinine, and albumin for inpatients who receive dialysis or other specialized interventions, allowing us to analyze detailed lab information for patients with dialysis-requiring AKI [22, 23].

We identified new-onset AKI-D cases between 1 January 2015 and 31 December 2018. Clinical diagnoses in the NHIRD are coded using International Classification of Diseases, 9th/10th Revision, Clinical Modification (ICD-9/10-CM) codes (Supplementary data, Table S1). AKI-D patients were defined as those without a prior end-stage kidney disease (ESKD) diagnosis who underwent acute dialysis during the index hospitalization [24]. Although our claims database does not systematically document the precise clinical indications for dialysis in each case, acute dialysis is typically initiated for volume overload not responsive to medical therapy, refractory hyperkalemia or severe metabolic derangements. The exclusion criteria were individuals aged <18 years, those who died or required re-dialysis within 90 days post-discharge, and those lacking repeated sCr measurements during hospitalization, within 90 days post-discharge, and between 90 days and 180 days post-discharge. In addition, patients with a history of kidney transplantation were also excluded. Finally, we included only those AKI-D patients who survived beyond 90 days after discontinuing dialysis, to focus on “AKD survivors” [25].

The study protocol received approval from the Institutional Review Board (IRB) of the National Research Program for Biopharmaceuticals Institutional Review Board (NRPB2014050014) and the IRB of the National Health Research Institutes (EC1060402-E). As patient identification numbers are encrypted in the NHIRD and individual identification is impossible, the need for informed consent was waived (http://doi.org/10.6084/m9.figshare.21731012).

### Definitions

Baseline sCr was defined as the nadir value obtained after the preceding admission for those with more than one admission within 1 year before the index admission. For individuals without a previous admission, baseline sCr was defined as the mean outpatient value over the 180 days before the index admission [26, 27]. CKD was defined as kidney structure or function abnormalities lasting more than 3 months and was classified into five categories following the KDIGO consensus [28]. We refer to the period from dialysis discontinuation up to 90 days as AKD [25].

### Covariates included in the analysis

We identified preadmission comorbidities within a 1-year period before the index hospitalization to reduce selection bias [29], requiring at least one inpatient admission or three outpatient visits for each condition [20]. Comorbidities, including hypertension, diabetes, myocardial infarction, heart failure, cerebrovascular disease, chronic liver disease, chronic obstructive pulmonary disease and malignancies were identified using ICD-9/10-CM codes (Supplementary data, Table S1). Baseline laboratory data were collected within 3 months following the discontinuation of RRT, using the initial test results when multiple results were available. Medications used within 180 days prior to the index hospitalization were considered co-existing medications. Information regarding the utilization of antiplatelet agents, statins, urate-lowering medications, alpha-blockers, beta-blockers, angiotensin-converting enzyme inhibitors (ACEIs), angiotensin receptor blockers (ARBs), mineralocorticoid receptor antagonists and calcium channel blockers was systematically documented (Supplementary data, Table S2). The Charlson Comorbidity Index (CCI) was used to assess disease severity [30]. Hospitalization procedures affecting kidney function, such as mechanical ventilation, coronary artery bypass grafting (CABG), percutaneous transluminal coronary angioplasty (PTCA), intra-aortic balloon pump insertion, extracorporeal membrane oxygenation (ECMO) and major surgical interventions were also recorded.

### Outcome assessments

The primary outcome was the incidence of major adverse kidney events (MAKEs) after discontinuation of renal replacement therapy (RRT), with secondary outcomes being all-cause mortality and major adverse cardiovascular events (MACEs). The index date was defined as 90 days after dialysis discontinuation, in line with the definition of AKD survivors [31]. Participants were monitored from 1 January 2015 to 31 December 2018, or until an event or death. MAKEs included CKD progression, re-dialysis and all-cause mortality, while MACEs encompassed coronary artery disease, heart failure, stroke and mortality [32]. CKD progression was defined as a persistent doubling of sCr from post-AKI-D baseline for at least 3 months or the initiation of maintenance dialysis (Fig. 1).

### Statistical analysis

Baseline differences among UCR trajectory groups were analyzed using descriptive statistics. Continuous data were expressed as mean ± standard deviation, and categorical data as number (percentage). Comparisons were made using the X² or Fishe's exact test, with normality assessed by the Kolmogorov–Smirnov test. UCR levels were reevaluated every 2 weeks for 90 days, and participants were categorized into longitudinal trajectory groups using GBTM, selected based on Bayesian information criterion (BIC) and Akaike information criterion (AIC) (Supplementary Appendix). Continuous variables and normally distributed data were compared using one-way analysis of variance, while the Kruskal–Wallis test was used for non-normally distributed data. UCR trajectory distributions were visualized using Manhattan plots, Uniform Manifold Approximation and Projection (UMAP) analysis and Sankey diagrams, with key features determined by a standardized mean difference >0.3 [33, 34]. We fit multivariable Cox proportional hazards models to estimate hazard ratios (HRs) for primary and secondary outcomes, after confirming no violation of the proportional hazards assumption using Schoenfeld residuals. Subgroup analyses were conducted to assess the effects of age, sex, comorbidities and medication history [35]. Different models were used to validate the study results, including eligibility with propensity score for multiple treatments, Cox regression with different covariates and overlap weighting with different sampling populations. Specificity analyses were conducted to investigate the relationships between trajectory groups and four independent events (lung cancer, pneumonia, traffic accidents and deafness) and to evaluate potentially unmeasured confounding. All analyses were performed using Stata/MP version 16 (StataCorp, College Station, TX, USA), SAS version 9.2 (SAS Inc., Cary, NC, USA), and R software, version 3.2.2 (Free Software Foundation, Inc., Boston, MA, USA) [36].

## RESULTS

### Characteristics of the study population

A total of 9717 AKD survivors were included, with a mean age of 66.1 years, and 5799 (59.7%) male. Over an average follow-up of 1.2 years, 43.7% (4244/9717) experienced MAKEs, 2553 patients (26.3%) died, 3024 patients (31.1%) developed MACEs and 1587 patients (16.3%) developed ESKD. AKI was attributed to sepsis in 4164 patients (42.9%). Hypertension was the leading comorbidity with a prevalent in 6090 (62.7%) patients, while diabetes was present in 3555 (36.6%) patients (**Table 1**). Patients with MAKEs were older, had higher comorbidity burdens and underwent more interventions during hospitalization. Subsequent to discharge, individuals who experienced MAKEs exhibited lower levels of UCR and albumin during their initial follow-up visit. Additionally, they underwent a higher frequency of follow-up visits within the nephrology department and experienced a higher incidence of all-cause mortality and a lower incidence of MACEs.

**Table 1:** Baseline characteristics of study participants categorized according to the occurrence of MAKEs.

	**Total patient (*n* = 9717)**	**MAKE (*n* = 4244)**	**No MAKE (*n* = 5473)**	***P***
Demographic factors, mean ± SD or *n* (%)				
Age, years	66.1 ± 15.9	69.5 ± 14.4	63.4 ± 16.5	<.001
Gender (male)	5799 (59.7)	2448 (57.7)	3351 (61.2)	<.001
CCI score	3.2 ± 2.4	4.0 ± 2.4	2.6 ± 2.3	<.001
Hypertension	6090 (62.7)	3024 (71.3)	3066 (56.0)	<.001
Diabetes mellitus	3555 (36.6)	1774 (41.8)	1781 (32.5)	<.001
Baseline kidney function, *n* (%)				<.001
CKD stage 0–2	3540 (36.4)	956 (22.5)	2584 (47.2)	
CKD stage 3	2590 (26.7)	1006 (23.7)	1584 (28.9)	
CKD stage 4	1911 (19.7)	1117 (26.3)	794 (14.5)	
CKD stage 5	1676 (17.3)	1165 (27.5)	511 (9.3)	
Intervention or complication during index hospitalization, mean ± SD or *n* (%)				
Hospital length of stay, days	28.2 ± 26.3	28.9 ± 26.3	27.6 ± 26.3	.019
ICU admission	7123 (73.3)	2780 (65.5)	4343 (79.4)	<.001
MV	8921 (91.8)	3869 (91.2)	5052 (92.3)	.042
Prolonged MV ≥4 days	4239 (43.6)	1519 (35.8)	2720 (49.7)	<.001
Re-intubation	254 (2.6)	98 (2.3)	156 (2.9)	.097
Pleural effusion	360 (3.7)	194 (4.6)	166 (3.0)	<.001
Chest tube	398 (4.1)	146 (3.4)	252 (4.6)	.004
ARDS	530 (5.5)	234 (5.5)	296 (5.4)	.821
CABG	181 (1.9)	60 (1.4)	121 (2.2)	.004
PTCA	581 (6.0)	304 (7.2)	277 (5.1)	<.001
IABP	273 (2.8)	83 (2.0)	190 (3.5)	<.001
ECMO	252 (2.6)	49 (1.2)	203 (3.7)	<.001
Cardiac surgery	714 (7.4)	185 (4.4)	529 (9.7)	<.001
GI surgery	208 (2.1)	88 (2.1)	120 (2.2)	.688
AKI contributors, *n* (%)				
Sepsis	4164 (42.9)	1592 (37.5)	2572 (47.0)	<.001
Hypovolemic shock	295 (3.0)	128 (3.0)	167 (3.1)	.920
Heart failure	1466 (15.1)	789 (18.6)	677 (12.4)	<.001
CT with contrast	2451 (25.2)	908 (21.4)	1543 (28.2)	<.001
Other or mixed etiology	1341 (13.8)	827 (9.5)	514 (15.7)	<.001
Medication before index hospitalization, *n* (%)				
Antiplatelet	1021 (10.5)	525 (12.4)	496 (9.1)	<.001
Statin	3155 (32.5)	1483 (34.9)	1672 (30.6)	<.001
Urate-lowering drug	2903 (29.9)	1521 (35.8)	1382 (25.3)	<.001
Alpha-blocker	160 (1.7)	98e (2.3)	62 (1.1)	<.001
Beta-blocker	978 (10.1)	509 (12.0)	469 (8.6)	<.001
ACEI or ARB	628 (6.5)	326 (7.7)	302 (5.5)	<.001
MRA	320 (3.3)	101 (2.4)	184 (3.4)	.666
Diuretics	5166 (53.2)	2350 (42.9)	2816 (66.4)	<.001
CCB	1301 (13.4)	720 (17.0)	581 (10.6)	<.001
Other anti-hypertensives	176 (1.8)	63 (2.1)	75 (1.4)	<.001
First follow-up laboratory results after discharge				
UCR	19.3 ± 12.1	19.0 ± 13.0	19.6 ± 11.4	.018
Albumin, g/dL	3.2 ± 0.5	3.2 ± 0.5	3.3 ± 0.5	<.001
Number of follow-up visits within 3 months after discharge				
Nephrology department	1.6 ± 2.0	2.0 ± 2.1	1.3 ± 1.8	<.001
General physician department	0.7 ± 1.9	0.7 ± 1.9	0.7 ± 1.8	.683
Outcome, *n* (%)				
All-cause mortality	2553 (26.3)	2553 (60.2)	0 (0.0)	<.001
MACEs	3024 (31.1)	217 (5.1)	2807 (51.3)	<.001
ESKD	1587 (16.3)	1587 (37.4)	0 (0.0)	<.001
Re-admission	5925 (61.0)	3557 (79.1)	2568 (46.9)	<.001

ARDS, acute respiratory distress syndrome; CCB, calcium channel blocker; COPD, chronic obstructive pulmonary disease; CT, computerized tomography; GI, gastrointestinal; IABP, intra-aortic balloon pump; MRA, mineralocorticoid receptor antagonists; MV, mechanical ventilation; SD, standard deviation.

### Identification of UCR trajectories using the GBTM

**Figure 2:**
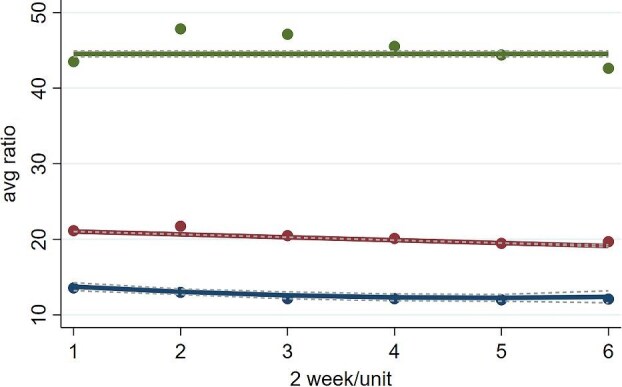
Three groups were identified based on trajectories of the mean values of UCR every 2 weeks after weaning off RRT. The blue line represents Group 1, which demonstrates a downward trend initially, showing a gradual decrease in the average UCR over the first 8 weeks, followed by a slight increase in the subsequent 4 weeks. The red line represents Group 2, which displays a consistent, gradual decline in the average UCR across the entire 12-week period. The green line represents Group 3, which remains relatively stable, maintaining a consistent average UCR over the 12-week period. Solid lines indicate the observed values, while dotted lines represent the fitted trajectories along with their 95% CIs.

The GBTM algorithm identified distinct UCR trajectories ranging from two-group to four-group models, with different degrees of polynomials in each group. The selection of the three-group model was substantiated by its excellent balance between complexity and simplicity, which was evident from the lowest BIC values and substantial AIC reduction (Supplementary data, Table S3) [37, 38]. The results of the GBTM are shown in Fig. 2. In the chosen three-group model, with quadratic specifications for all groups, we defined the following trajectories: a low trajectory for Group 1 (*n* = 1822, 18.8%), a medium trajectory for Group 2 (*n* = 7065, 72.7%) and a high trajectory for Group 3 (*n* = 830, 8.5%). This model was not just coherent in aligning the estimated probabilities of group membership with the actual distribution of study members but also excelled in classification accuracy. This is evidenced by the high average posterior probabilities for group membership alongside a satisfactory percentage for the smallest group.

### Characteristics of the three UCR trajectory groups

The three UCR trajectory groups showed statistically significant differences in mean UCR at every timepoint up to 90 days following the discontinuation of RRT (Fig. 2). In Group 1, the UCR trajectory showed a quadratic curve (second-order) trajectory, with a steady and gradual decrease in mean UCR over the first 8-week period, followed by a gradual slight increase over the remaining 4 weeks. Group 2 exhibited a linear curve (first-order) trajectory, showing a steady and gradual decrease in mean UCR over the 12-week period. On the other hand, Group 3 followed a zero-order curve (intercept), indicating a consistent mean UCR value over the 12-week period.

Table 2 presents the characteristics of the three trajectory groups, which differed significantly in their clinical features. The initial post-discharge UCR levels were 10.7, 19.3 and 38.4 for the trajectory Groups 1, 2 and 3, respectively. Greater proportions of elderly and female patients were found in the high UCR trajectory group (Group 3). The average CCI score and prevalence of hypertension and advanced CKD exhibited a distinct downward trend across the three UCR trajectory groups, from Group 1 (low) to Group 3 (high).

**Table 2:** Baseline characteristics of enrollee according to different UCR trajectory groups.

	**Total patient (*n* = 9717)**	**Group 1 (*n* = 1822)**	**Group 2 (*n* = 7065)**	**Group 3 (*n* = 830)**	***P*** **^a^**
UCR	19.3 ± 12.1	10.7 ± 8.1	19.3 ± 9.4	38.4 ± 17.6	<.001
Demographic factors					
Age, years	66.1 ± 15.9	62.6 ± 15.6	66.5 ± 15.7	70.5 ± 16.3	<.001
Gender (male), *n* (%)	5799 (59.7)	1211 (66.5)	4236 (60.0)	352 (42.4)	<.001
CCI score	3.2 ± 2.4	3.6 ± 2.3	3.2 ± 2.5	2.7 ± 2.4	<.001
Hypertension, *n* (%)	6090 (62.7)	1229 (67.5)	4354 (61.6)	507 (61.1)	<.001
Diabetes mellitus, *n* (%)	3555 (36.6)	685 (37.6)	2603 (36.8)	267 (32.2)	.019
Baseline kidney function, *n* (%)					<.001
CKD stage 0–2	3540 (36.4)	332 (18.2)	2697 (38.2)	511 (61.6)	
CKD stage 3	2590 (26.7)	293 (16.1)	2047 (29.0)	250 (30.1)	
CKD stage 4	1911 (19.7)	367 (20.1)	1491 (21.1)	53 (6.4)	
CKD stage 5	1676 (17.3)	830 (45.6)	830 (11.8)	16 (1.9)	
Intervention or complication during index hospitalization, mean ± SD or *n* (%)					
Hospital length of stay, days	28.2 ± 26.3	23.4 ± 24.8	27.6 ± 25.5	43.9 ± 30.4	<.001
ICU admission	7123 (73.3)	921 (50.6)	5446 (77.1)	756 (91.1)	<.001
MV	8921 (91.8)	1552 (85.2)	6547 (92.7)	822 (99.0)	<.001
Prolonged MV ≥4 days	4239 (43.6)	415 (22.8)	3178 (45.0)	646 (77.8)	<.001
Re-intubation	254 (2.6)	21 (1.2)	177 (2.5)	56 (6.8)	<.001
Pleural effusion	360 (3.7)	46 (2.5)	254 (3.6)	60 (7.2)	<.001
Chest tube	398 (4.1)	36 (2.0)	280 (4.0)	82 (10.0)	<.001
ARDS	530 (5.5)	61 (3.4)	374 (5.3)	95 (11.5)	<.001
CABG	181 (1.9)	18 (1.0)	148 (2.1)	15 (1.8)	.008
PTCA	581 (6.0)	116 (6.4)	429 (6.1)	36 (4.3)	.102
IABP	273 (2.8)	30 (1.7)	210 (3.0)	33 (4.0)	.001
ECMO	252 (2.6)	23 (1.3)	169 (2.4)	60 (7.2)	<.001
Cardiac surgery	714 (7.4)	67 (3.7)	532 (7.5)	115 (13.9)	<.001
GI surgery	208 (2.1)	21 (1.2)	152 (2.2)	35 (4.2)	<.001
AKI contributors, *n* (%)					
Sepsis	4164 (42.9)	507 (27.8)	3105 (44.0)	552 (66.5)	<.001
Hypovolemic shock	295 (3.0)	25 (1.4)	233 (3.3)	37 (4.5)	<.001
CT with contrast	2451 (25.2)	340 (18.7)	1829 (25.9)	282 (34.0)	<.001
Congestive heart failure	1466 (15.1)	215 (11.8)	1092 (15.5)	159 (19.2)	<.001
Other or mixed etiology	2807 (28.9)	950 (52.1)	1898 (26.9)	0 (0.0)	<.001
Medication before index hospitalization, *n* (%)					
Antiplatelet	1021 (10.5)	147 (8.1)	764 (10.8)	110 (13.3)	<.001
Statin	3155 (32.5)	648 (35.6)	2274 (32.2)	233 (28.1)	<.001
Urate-lowering drug	2903 (29.9)	650 (35.7)	2087 (29.5)	166 (20.0)	<.001
Alpha-blocker	160 (1.7)	36 (2.0)	117 (1.7)	7 (0.8)	.104
Beta-blocker	978 (10.1)	238 (13.1)	688 (9.7)	52 (6.3)	<.001
ACEI or ARB	628 (6.5)	111 (6.1)	468 (6.6)	49 (5.9)	.563
MRA	320 (3.3)	37 (2.0)	252 (3.6)	31 (3.7)	.004
CCB	1301 (13.4)	306 (16.8)	893 (12.6)	102 (12.3)	<.001
Diuretics	5166 (53.2)	1022 (56.1)	3685 (52.2)	459 (55.3)	.005
Other anti-hypertensives	176 (1.8)	41 (2.3)	125 (1.8)	10 (1.2)	.153
First follow-up laboratory results after discharge					
UCR	19.3 ± 12.1	10.7 ± 8.1	19.3 ± 9.4	38.4 ± 17.6	<.001
Albumin, g/dL	3.2 ± 0.5	3.4 ± 0.5	3.2 ± 0.5	2.9 ± 0.5	<.001
Number of follow-up visits within 3 months after discharge					
Nephrology department	1.6 ± 2.0	2.8 ± 2.2	1.4 ± 1.8	0.5 ± 1.2	<.001
General physician department	0.7 ± 1.9	0.7 ± 2.0	0.7 ± 1.9	0.5 ± 1.5	.002
Outcome, *n* (%)					
MAKEs	4244 (43.7)	1026 (56.3)	2823 (40.0)	395 (47.6)	<.001
All-cause mortality	2553 (26.3)	341 (18.7)	1853 (26.2)	359 (43.3)	<.001
MACEs	3024 (31.1)	473 (26.0)	2165 (30.6)	386 (46.5)	<.001
ESKD	1587 (16.3)	703 (38.6)	864 (12.2)	20 (2.4)	<.001
Re-admission	5925 (61.0)	1129 (62.0)	4303 (60.9)	493 (59.4)	.442

a Analysis of variance.

ARDS, acute respiratory distress syndrome; CCB, calcium channel blocker; COPD, chronic obstructive pulmonary disease; CT, computerized tomography; GI, gastrointestinal; MRA, mineralocorticoid receptor antagonists; MV, mechanical ventilation; SD, standard deviation.

Prior to the index hospitalization, the prevalence of medication use varied significantly among the trajectory groups. Group 3 were more likely to use anti-platelets and mineralocorticoid receptor antagonist medications, but less likely to use statins, urate-lowering agents, beta-blockers and calcium channel blockers than Groups 1 and 2. During the index hospitalization, there were noticeable upward gradients for hospital length of stay and the incidence of intensive care unit (ICU) admissions from Group 1 to Group 3. Statistically significant associations were identified for the incidence of receiving interventions, and Group 3 were more likely to receive various interventions except CABG and PTCA.

### Outcomes of interest

Within 3 months after discharge, Group 3 had the lowest albumin levels, as well as the fewest follow-up visits at nephrology and general physician departments. After 90 days of discontinuing RRT, Group 2 had the lowest incidence of MAKEs. Group 1 had the highest incidence of MAKEs but the lowest incidence of mortality and MACEs. In contrast, Group 3 had the highest incidence of mortality and MACEs.

**Figure 3:**
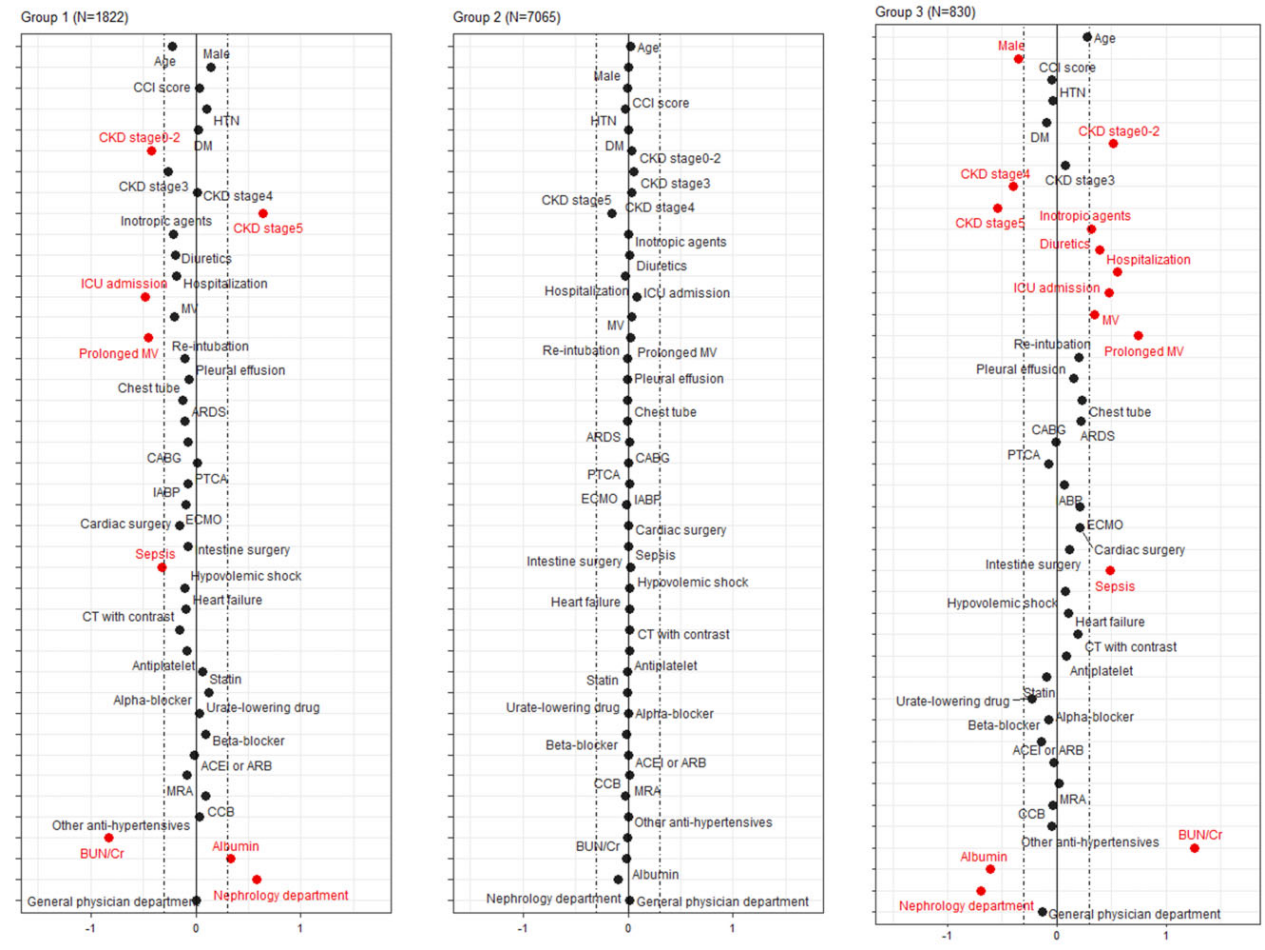
Manhattan plot of the standardized differences across the three UCR trajectory groups. This figure depicts the clinical characteristics of the three trajectory groups stratified by UCR levels. Group 1 (*N* = 1822) includes patients with the most advanced stages of CKD, the lowest UCR levels, and the fewest instances of sepsis, ICU admissions, and extended mechanical ventilation. Group 2 (*N* = 7065) represents patients with intermediate characteristics. Group 3 (*N* = 830) consists of more female patients and those with the best renal function, but the lowest albumin levels. This group also experienced the highest incidence of sepsis, longer hospital stays, more frequent ICU admissions and a higher frequency of receiving interventions. Solid black dots indicate observed values, red dots denote significant differences, and dashed lines represent 95% CIs. The y-axis shows the standardized difference value, and the x-axis represents the s parameters. The dashed-dotted vertical lines represent standardized difference cutoffs of >0.3 or <−0.3. ARDS, acute respiratory distress syndrome; CCB, calcium channel blocker; COPD, chronic obstructive pulmonary disease; CT, computerized tomography; IABP, intra-aortic balloon pump; MRA, mineralocorticoid receptor antagonists.

The key features for each trajectory group were depicted in Fig. 3. Group 1 had the most advanced CKD, the lowest UCR levels, and the fewest sepsis cases, ICU admissions and cases of prolonged mechanical ventilation. In contrast, Group 3 comprised more female patients and presented with the best kidney function, the lowest albumin levels, the most sepsis cases, longer hospital stays, more ICU admissions and a higher frequency of receiving interventions. UMAP also revealed significant differences among the three groups (Supplementary data, Fig. S1).

### UCR trajectories predicted incident MAKEs in the AKD survivors

**Figure 4:**
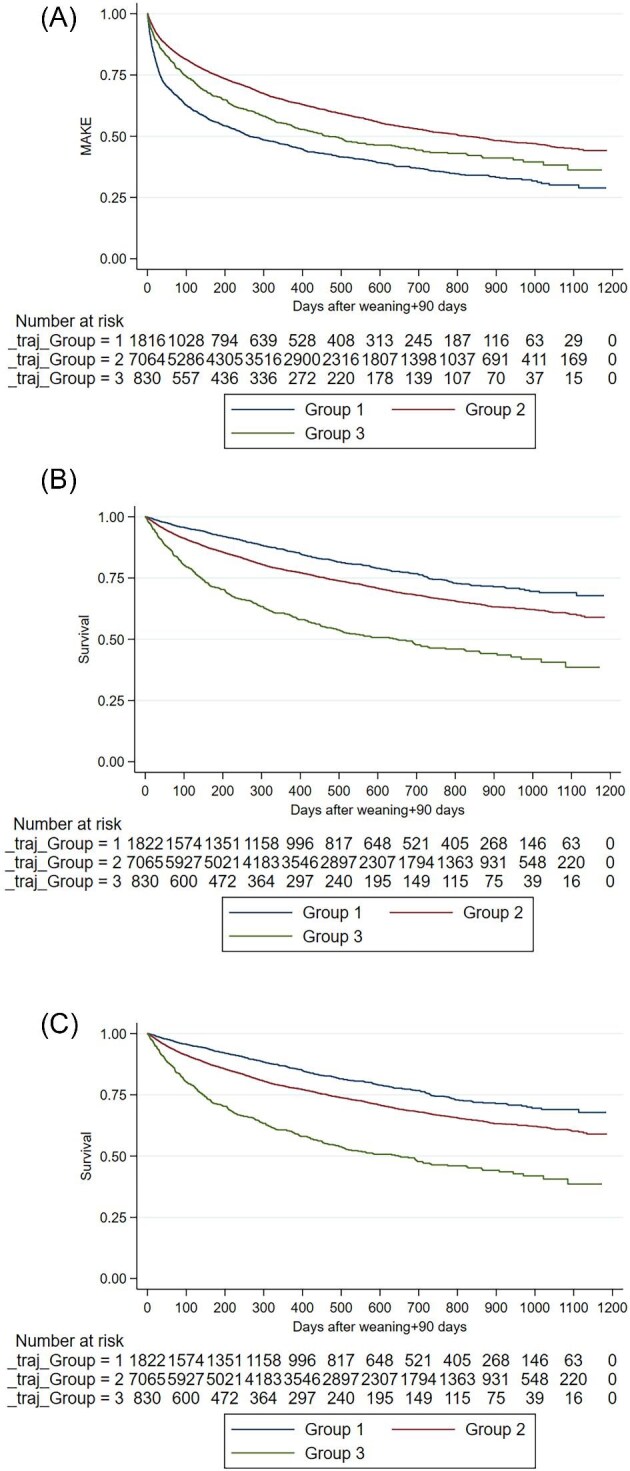
The follow-up period was 1200 days for adverse outcomes among the three UCR trajectory groups using the Kaplan–Meier method after balancing covariates with overlap weighting. (**A**) MAKEs: Group 1 (blue line) had the highest incidence of MAKEs, followed by Group 3 (green line) and Group 2 (red line). (**B**) All-cause mortality: Group 1 (blue line) exhibited the highest survival rates, followed by Group 2 (red line) and Group 3 (green line), which had the lowest survival rates. (**C**) MACEs: Group 1 (blue line) showed the best outcomes, with the lowest incidence of MACEs, followed by Group 2 (red line) and Group 3 (green line).

Among the 9717 enrolled patients, 43.7% (4244) developed 90-day MAKEs. Kaplan–Meier plots showed significant associations between UCR trajectories and MAKEs (log rank *P* < .001, Fig. 4A). Cox proportional hazards analysis identified age [HR 1.01, 95% confidence interval (CI) 1.01–1.01], male gender (HR 1.10) and high CCI score (HR 1.12) as risk factors for MAKEs, while ICU admission (HR 0.86), cardiac surgery (HR 0.72) and higher albumin with 3 months after discharge (HR 0.70) reduced risk. Advanced CKD stages significantly increased risks, particularly stage 5 (HR 2.88). Group 3 (HR 1.54) and Group 1 (HR 1.30) had higher 90-day MAKEs risk compared with Group 2 (Table 3).

**Table 3:** Cox proportional hazards models depicting the association between different variables and the risk of MAKEs in the study population.

**Predictors**	**HR (95% CI)**	***P*-value**
Demographic factors		
Age, years	1.01 (1.01–1.01)	<.001
Male gender	1.10 (1.03–1.18)	.003
CCI score	1.12 (1.11–1.14)	<.001
Hypertension	1.10 (1.02–1.18)	.016
Diabetes mellitus	0.92 (0.87–0.98)	.015
Baseline kidney function (reference: CKD stage 0–2)		
CKD stage 3	1.16 (1.06–1.28)	.001
CKD stage 4	1.86 (1.69–2.05)	<.001
CKD stage 5	2.88 (2.60–3.18)	<.001
Intervention or complication during index hospitalization		
Hospital length of stay, days	1.005 (1.004–1.006)	<.001
ICU admission	0.86 (0.79–0.93)	<.001
MV	0.93 (0.83–1.04)	.198
Prolonged MV ≥4 days	0.93 (0.85–1.02)	.119
Re-intubation	1.12 (0.91–1.38)	.293
Pleural effusion	1.36 (1.17–1.58)	<.001
Chest tube	1.06 (0.89–1.26)	.509
ARDS	1.07 (0.92–1.23)	.388
CABG	1.05 (0.75–1.46)	.780
PTCA	1.21 (1.05–1.39)	.010
IABP	0.81 (0.64–1.03)	.088
ECMO	0.98 (0.69–1.38)	.891
Cardiac surgery	0.72 (0.57–0.90)	.004
GI surgery	1.33 (1.07–1.65)	.009
AKI contributors		
Sepsis	0.86 (0.80–0.92)	<.001
Hypovolemic shock	0.93 (0.77–1.11)	.423
CT with contrast	0.85 (0.78–0.92)	<.001
Medication before index hospitalization		
Antiplatelet	1.09 (0.99–1.20)	.081
Statin	0.90 (0.84–0.97)	.006
Urate-lowering drug	1.07 (0.99–1.15)	.082
Alpha-blocker	1.04 (0.85–1.28)	.703
Beta-blocker	1.02 (0.92–1.13)	.693
ACEI or ARB	1.05 (0.93–1.18)	.435
MRA	0.97 (0.82–1.16)	.773
CCB	1.05 (0.96–1.14)	.311
Other anti-hypertensives	0.96 (0.78–1.18)	.715
Laboratory results within 3 months after discharge		
Albumin, g/dL	0.70 (0.65–0.74)	<.001
Number of follow-up visits within 3 months after discharge		
Nephrology department	1.02 (1.001–1.04)	.038
General physician department	0.99 (0.98–1.01)	.377
UCR trajectory groups (reference: Group 2)		
Group 1	1.30 (1.20–1.41)	<.001
Group 3	1.54 (1.38–1.73)	<.001

Model adjustment: all HRs in this table were derived from a multivariable-adjusted Cox proportional hazards model. The following covariates were included in the model: demographic factors (age, sex), comorbidities (e.g. CCI, hypertension, diabetes), baseline kidney function (CKD stage), interventions or complications during the index hospitalization (MV, cardiac surgery, etc.), preadmission medications (e.g. antiplatelet agents, beta-blockers), albumin within 3 months post-discharge, number of follow-up visits in nephrology and general physician clinics, and the UCR trajectory groups.

ARDS, acute respiratory distress syndrome; CCB, calcium channel blocker; COPD, chronic obstructive pulmonary disease; CT, computerized tomography; GI, gastrointestinal; MRA, mineralocorticoid receptor antagonists; MV, mechanical ventilation.

### UCR trajectories predicted mortality and incident MACEs in the AKD survivors

During follow-up, 26.3% (2553/9717) died. Mortality risks were stratified by UCR groups (log rank *P* < .001, Fig. 4B), with Group 3 having the highest risk (HR 1.59) and Group 1 the lowest (HR 0.84). Key risk factors for mortality included age, male gender, high CCI and extended hospital stays, while cardiac surgery and higher albumin reduced mortality risk (Table 4).

**Table 4:** Cox proportional hazards models depicting the association between different variables and the risk of mortality in the study population.

**Predictors**	**HR (95% CI)**	***P*-value**
Demographic factors		
Age, years	1.03 (1.03–1.04)	<.001
Male gender	1.18 (1.08–1.28)	<.001
CCI score	1.15 (1.13–1.17)	<.001
Hypertension	0.92 (0.84–1.01)	0.079
Diabetes mellitus	0.90 (0.82–0.98)	0.014
Baseline kidney function (reference: CKD stage 0–2)		
CKD stage 3	1.07 (0.96–1.18)	0.240
CKD stage 4	1.25 (1.11–1.40)	<.001
CKD stage 5	1.01 (0.87–1.17)	0.918
Intervention or complication during index hospitalization		
Hospital length of stay, days	1.004 (1.003–1.01)	<.001
ICU admission	0.97 (0.87–1.32)	0.621
MV	1.18 (0.99–1.41)	0.072
Prolonged MV >4 days	0.93 (0.84–1.04)	0.198
Re-intubation	1.19 (0.95–1.49)	0.141
Pleural effusion	1.32 (1.10–1.59)	0.003
Chest tube	1.07 (0.87–1.32)	0.514
ARDS	0.97 (0.82–1.14)	0.695
CABG	0.68 (0.42–1.12)	0.129
PTCA	1.29 (1.07–1.55)	0.007
IABP	0.80 (0.60–1.08)	0.145
ECMO	1.29 (0.84–1.98)	0.254
Cardiac surgery	0.63 (0.47–0.83)	0.001
GI surgery	1.08 (0.82–1.41)	0.586
AKI contributors		
Sepsis	0.999 (0.92–1.09)	0.986
Hypovolemic shock	0.94 (0.77–1.16)	0.562
CT with contrast	0.86 (0.78–0.96)	0.006
Medication before index hospitalization		
Antiplatelet	1.18 (1.05–1.33)	0.006
Statin	0.78 (0.70–0.85)	<.001
Urate-lowering drug	1.00 (0.91–1.10)	0.999
Alpha-blocker	1.29 (1.001–1.67)	0.049
Beta-blocker	0.86 (0.75–0.99)	0.035
ACEI or ARB	1.07 (0.92–1.25)	0.367
MRA	0.99 (0.79–1.23)	0.893
CCB	1.13 (1.01–1.26)	0.035
Other anti-hypertensives	0.89 (0.67–1.18)	0.427
Laboratory results within 3 months after discharge		
Albumin, g/dL	0.54 (0.50–0.58)	<.001
Number of follow-up visits within 3 months after discharge		
Nephrology department	0.93 (0.91–0.96)	<.001
General physician department	1.00 (0.98–1.02)	0.988
UCR trajectory groups (reference: Group 2)		
Group 1	0.84 (0.74–0.95)	0.007
Group 3	1.59 (1.41–1.80)	<.001

Model adjustment: all HRs in this table were derived from a multivariable-adjusted Cox proportional hazards model. The following covariates were included in the model: demographic factors (age, sex), comorbidities (e.g. CCI, hypertension, diabetes), baseline kidney function (CKD stage), interventions or complications during the index hospitalization (MV, cardiac surgery, etc.), preadmission medications (e.g. antiplatelet agents, beta-blockers), albumin within 3 months post-discharge, number of follow-up visits in nephrology and general physician clinics, and the UCR trajectory groups.

ARDS, acute respiratory distress syndrome; CCB, calcium channel blocker; COPD, chronic obstructive pulmonary disease; CT, computerized tomography; GI, gastrointestinal; MRA, mineralocorticoid receptor antagonists; MV, mechanical ventilation.

Overall, 31.1% (3024) experienced MACEs. Groups 1, 2 and 3 were associated with low, medium, and high risks of MACEs, respectively (log rank *P* < .001, Fig. 4C). Group 3 had the highest risk of MACEs (HR 1.57), while factors like age, male gender, high CCI, PTCA, and antiplatelet or calcium channel blockers increased MACEs risk. Additionally, patients with advanced chronic kidney disease, particularly those in stages 4 and 5, face a markedly higher risk, with HRs of 1.30 and 1.15. In contrast, statins and higher albumin reduced MACEs risk (Table 5). Sankey diagrams showed UCR trajectories predicted kidney but not cardiovascular outcomes Supplementary data, Fig. S2.

**Table 5:** Cox proportional hazards models depicting the association between different variables and the risk of MACEs in the study population.

**Predictors**	**HR (95% CI)**	***P*-value**
Demographic factors		
Age, years	1.03 (1.02–1.03)	<.001
Male gender	1.18 (1.09–1.27)	<.001
CCI score	1.12 (1.11–1.14)	<.001
Hypertension	0.97 (0.89–1.05)	.443
Diabetes mellitus	0.92 (0.85–1.00)	.043
Baseline kidney function (reference: CKD stage 0–2)		
CKD stage 3	1.10 (0.99–1.21)	.069
CKD stage 4	1.30 (1.17–1.46)	<.001
CKD stage 5	1.15 (1.01–1.31)	.036
Intervention or complication during index hospitalization		
Hospital length of stay, days	1.004 (1.003–1.005)	<.001
ICU admission	1.006 (0.91–1.11)	.906
MV	1.15 (0.98–1.35)	.093
Prolonged MV ≥4 days	0.90 (0.81–0.99)	.035
Re-intubation	1.15 (0.93–1.43)	.200
Pleural effusion	1.25 (1.05–1.48)	.013
Chest tube	1.12 (0.93–1.36)	.243
ARDS	0.97 (0.83–1.13)	.691
CABG	0.68 (0.44–1.06)	.092
PTCA	1.55 (1.32–1.82)	<.001
IABP	1.01 (0.79–1.28)	.947
ECMO	1.29 (0.88–1.91)	.197
Cardiac surgery	0.59 (0.45–0.76)	<.001
GI surgery	1.15 (0.90–1.47)	.271
AKI contributors		
Sepsis	0.98 (0.90–1.06)	.567
Hypovolemic shock	0.97 (0.98–1.02)	.792
CT with contrast	0.86 (0.78–0.95)	.003
Medication before index hospitalization		
Antiplatelet	1.13 (1.01–1.26)	.029
Statin	0.85 (0.78–0.93)	<.001
Urate-lowering drug	1.02 (0.93–1.11)	.668
Alpha-blocker	1.11 (0.87–1.42)	.389
Beta-blocker	0.93 (0.83–1.06)	.273
ACEI or ARB	0.98 (0.85–1.13)	.828
MRA	0.97 (0.79–1.19)	.780
CCB	1.14 (1.03–1.26)	.015
Other anti-hypertensives	0.87 (0.67–1.12)	.283
Laboratory results within 3 months after discharge		
Albumin, g/dL	0.60 (0.56–0.65)	<.001
Number of follow-up visits within 3 months after discharge7		
Nephrology department	0.96 (0.94–0.98)	<.001
General physician department	1.003 (0.98–1.02)	.792
UCR Trajectory groups (reference: Group 2)		
Group 1	0.93 (0.83–1.04)	.194
Group 3	1.57 (1.40–1.77)	<.001

Model adjustment: all HRs in this table were derived from a multivariable-adjusted Cox proportional hazards model. The following covariates were included in the model: demographic factors (age, sex), comorbidities (e.g. CCI, hypertension, diabetes), baseline kidney function (CKD stage), interventions or complications during the index hospitalization (MV, cardiac surgery, etc.), preadmission medications (e.g. antiplatelet agents, beta-blockers), albumin within 3 months post-discharge, number of follow-up visits in nephrology and general physician clinics, and the UCR trajectory groups.

ARDS, acute respiratory distress syndrome; CCB, calcium channel blocker; COPD, chronic obstructive pulmonary disease; CT, computerized tomography; GI, gastrointestinal; MRA, mineralocorticoid receptor antagonists; MV, mechanical ventilation.

### Subgroup and sensitivity analyses

In subgroup analyses, Groups 1 and 3 consistently showed increased MAKEs risk compared with Group 2 across factors like sex, comorbidities, and use of ACEIs or ARBs (Supplementary data, Fig. S3). Mortality risk was lower in Group 1 and higher in Group 3 compared with Group 2 across various factors (Supplementary data, Fig. S4). Group 3 had a significantly higher MACEs risk, while Group 1 showed no significant difference (Supplementary data, Fig. S5). Across all models, both Groups 1 and 3 showed an increased risk for MAKEs compared with Group 2, while Group 3 also had an increased risk for mortality, and Group 1 had a reduced mortality risk compared with Group 2 (Supplementary data, Tables S4 and S5). Only Group 3 showed an increased MACEs risk (Supplementary data, Table S6). Specificity analysis showed no differences in lung cancer, pneumonia, traffic accidents or deafness among groups (Supplementary data, Table S7). Results remained robust across models and variables.

## DISCUSSION

Our study demonstrated a high burden of adverse outcomes among AKD survivors, with 43.7% developing incident MAKEs during the study period. Specifically, the mortality rate was 26.3%, and the MACEs rate was 31.1%, findings that align with previous research [34]. By applying a GBTM algorithm, we identified three UCR trajectories that captured dynamic patterns of UCR over the initial 90-day AKD window, which were strongly predictive of MAKEs, mortality and MACEs.

Elderly patients, male gender, and those with higher CCI scores, hypertension and advanced CKD were more likely to develop MAKEs. The distinct UCR trajectories were associated with 90-day MAKEs, with UCR severity displaying a U-shaped pattern as a significant independent predictor for incident MAKEs. Furthermore, UCR trajectories exhibited a severity-dependent correlation with progressively significant increases in all-cause mortality and were also associated with a higher incidence of MACEs. Notably, despite having relatively preserved baseline kidney function, which is typically associated with better outcomes, patients with high UCR levels displayed the highest risk of MAKEs, mortality and MACEs. This highlights the critical importance of considering UCR trajectories and their distinct characteristics in predicting the prognosis of patients with AKD. Conversely, patients with the lowest UCR levels had higher risks of MAKEs but lower risks of mortality compared with those with medium UCR levels. Our findings underscore the necessity for a minimum 3-month follow-up assessment of UCR patterns after discharge, enabling more precise risk stratification and identification of diverse clinical outcomes in AKD survivors.

UCR is a simple surrogate marker that can reflect neurohormonal activity [39, 40]. The relationship between neurohormonal activity and AKI is complex, and AKI-induced neurohormonal dysregulation has been shown to contribute to adverse outcomes such as CKD, hypertension and cardiovascular diseases [41, 42]. Patients with AKI are particularly susceptible to renin–angiotensin–aldosterone system (RAAS) disruption, which can cause inadequate blood pressure control, cardiovascular disease, fluid imbalance, electrolyte abnormalities and CKD [42]. As the sympathetic nervous system and the RAAS become activated, urea excretion may be disproportionately reduced due to decreased glomerular filtration rate and increased urea absorption, while sCr continues to be freely filtered, thereby elevating UCR [8, 10, 43].

In addition, UCR serves as a surrogate measure of fluid status and metabolism. A higher UCR has been associated with decreased serum albumin, body mass index and waist circumference, and it has also been suggested as an index of malnutrition [44].

Beyond the acute phase of AKI, maintaining optimal hydration and adequate nutrition remains critical for better long-term outcomes [45, 46]. Prior studies have reported a link between elevated UCR and increased mortality rates in various clinical settings, including heart failure, cardiovascular diseases, CKD, ESKD and critically ill patients [47–50]. In the current study, we further demonstrated that a high UCR trajectory, as determined by GBTM, was also associated with a markedly increased risk of MAKEs, mortality and MACEs in AKD patients.

Little research has investigated the clinical interpretation of a decreased UCR. The literature suggests that a low BUN level may be associated with low protein intake, liver disease or overhydration, while a low UCR (<10) may be attributed to inflammation, oxidative stress and endothelial dysfunction [51, 52]. Shen *et al.* found that UCR had a U-shaped association with all-cause mortality in the general population, and that patients with an extremely low UCR (<10) had a higher risk of mortality than those with a UCR between 11.4 and 14.6 [52]. Interestingly, our findings showed that the low UCR trajectory group had a significantly higher risk of MAKEs yet a lower risk of mortality compared with the medium UCR trajectory group. The difference in mortality risk between the low UCR groups in our study and Shen *et al*.’s study may be because none of our low trajectory group had an extremely low mean UCR (<10) and they had the highest nutritional level in terms of albumin. In addition, the low UCR group had a significantly higher prevalence of advanced CKD (stage 3 to 5) at baseline than the medium UCR trajectory group. These findings are consistent with our previous investigation, which found that AKI patients with pre-existing CKD had a significantly lower risk of mortality than those without pre-existing CKD [4].

Overall, our study highlights the importance of UCR levels in predicting the prognosis of AKD survivors. Different clinical courses and prognoses are reflected in the varying trajectories identified through GBTM. Applying GBTM to classify UCR trajectory subphenotype during the AKD period appears to improve risk assessment, fostering the development of precision medicine and refining risk stratification for AKD patients. Further research is needed to determine whether modifying UCR levels through targeted interventions can mitigate the risk of adverse outcomes in AKD survivors.

### Strengths and limitations

A major strength of this study is the detailed analysis of UCR trajectories in a large cohort of AKD survivors, being the first to link distinct UCR patterns to adverse outcomes during the AKD period. While our study contributes significantly to the field, it has notable limitations. First, the homogeneity of the Asian cohort limits generalizability of the findings. Second, the retrospective design and irregular kidney parameter measurements may have introduced survivor bias, though the use of real-world data from a nationwide database adds relevance. Third, reliance on diagnostic codes could underestimate conditions not captured within the medical system, possibly introducing confounding factors, although specificity analysis supports the robustness of our findings. Fourth, we lacked precise data on the specific clinical indications leading to acute dialysis initiation, since the claims-based database does not routinely record variables such as exact fluid status, electrolyte values at the time of dialysis or the presence of other metabolic derangements. Consequently, we could not distinguish between different dialysis triggers (for example, refractory hyperkalemia versus acute volume overload) or assess the relative severity of these triggers. This limitation may reduce our ability to stratify subgroups of AKI-D patients with distinct pathophysiological profiles and could influence the generalizability of our findings. Nonetheless, given that acute dialysis typically aligns with widely recognized clinical thresholds, we believe our overall identification of AKI-D remains clinically relevant. Additionally, differences in comorbidities and medication use across UCR trajectory groups may have influenced outcomes, although adjustments and sensitivity analyses were performed to minimize confounders. Lastly, while GBTM offers intuitive visual summaries, it relies on assumptions that must be carefully considered.

## CONCLUSION

In summary, our study indicates that distinct UCR trajectories can independently predict the risk of adverse outcomes in individuals who have survived AKD. A high UCR trajectory in the AKD period was associated with increased risks of MAKEs, mortality and MACEs, while a low UCR trajectory was linked to a significantly higher risk of MAKEs but a lower mortality risk. These findings provide important clinical evidence for integrating UCR trajectory monitoring into the long-term outcomes of AKD survivors, aiming to enhance prognosis and guide personalized interventions.

## Supplementary Material

sfaf175_Supplemental_File

## Data Availability

The datasets used and/or analyzed during the current study are available from the corresponding author on reasonable request.
