# Supplementary material for: Trajectories of urea‑to‑creatinine ratio and risk of clinical outcomes in survivors of acute kidney disease: a population-based study
Source: Clin Kidney J. 2025 May 29;18(6):sfaf175. doi: 10.1093/ckj/sfaf175 (PMC12202997; doi:10.1093/ckj/sfaf175)
Supplement: sfaf175_Supplemental_File [file sfaf175_supplemental_file.pdf]

Supplemental Digital Content

Supplementary material and methods: Group-based trajectory model
Table S1. Diagnosis codes used in the study
Table S2. Medication codes used in this study
Table S3. Average posterior probabilities of group assignment and statistics of model fit
Table S4. Sensitivity analysis for risk of MAKEs among different trajectory groups
Table S5. Sensitivity analysis for risk of mortality among different trajectory groups
Table S6. Sensitivity analysis for risk of MACEs among different trajectory groups
Table S7. Specificity analysis for risk of 4 independent events, lung cancer, pneumonia, traffic accident, and deafness among different trajectory groups
Figure S1. UMAP visualization of three UCR trajectory groups
Figure S2. Sankey diagrams showing the natural course of three UCR trajectory groups. (A) Baseline CKD – post-AKD UCR – clinical kidney outcome; (B) Baseline CKD – post-AKD UCR – clinical cardiovascular outcome
Figure S3. Subgroup analysis of MAKEs
Figure S4. Subgroup analysis of mortality
Figure S5. Subgroup analysis of MACEs

Supplementary material and methods: Group-based trajectory model

The GBTM assigns individuals a likelihood of belonging to a particular group rather than strictly placing them in a specific group. The model connects baseline attributes to the probability of group association and addresses missing data under the assumption that data are missing at random. We tested multiple candidate models comprising different numbers of trajectory groups (two, three, or four) and varied polynomial orders (linear, quadratic, or cubic) to capture potential patterns in the UCR over the 90-day period. Model selection was guided primarily by the BIC while also considering the AIC. We aimed for a practical and parsimonious model that fit the data well, ensuring a close match between each group's estimated probability and the proportion of participants empirically assigned to that group. We further verified that each group's AvePP exceeded 0.7, that each group comprised at least 5% of the total population, and that the odds of correct classification based on posterior probabilities surpassed 5% [1, 2]. If needed, we reduced the polynomial degree (e.g., from cubic to quadratic) to ensure stable parameter estimates and avoid overfitting.

After comparing the fit statistics (BIC, AIC) and evaluating model parsimony, we selected a three-group model. This model yielded the most favorable BIC values and satisfied our criteria for AvePP, group size, and interpretability. The final model classified participants into three distinct UCR trajectory groups, each exhibiting different initial levels and slopes of UCR across the 90-day timeframe. Other criteria for model adequacy included maintaining sufficiently narrow confidence intervals for each trajectory group and assessing attrition to evaluate any bias arising from incomplete data.

In addition, we examined potential baseline covariates as time-invariant predictors of group membership, allowing us to explore how demographic and clinical characteristics might influence the probability of belonging to each trajectory group. We examined the relationships between trajectory groups and covariates using chi-square tests for proportions and Kruskal-Wallis tests for means. All tests were two-tailed, and statistical significance was set at $p < 0.05$.

In Table S3, we compare the two-, three-, and four-group solutions based on AvePP, BIC, AIC, and the smallest group proportion. The two-group model achieves a BIC of −110048.52 and an AIC of −110019.8, with a smallest group proportion of 9.3%. Although its AvePP values (0.9886 and 0.9335) suggest high classification accuracy, a two-group framework may provide relatively coarse discrimination of distinct UCR trajectories. The four-group model, meanwhile, indicates potential over-segmentation, as it attains a BIC of −107095.64 and an AIC of −107023.84, but the smallest group proportion drops to 1.0%, raising concerns about model parsimony and the clinical relevance of that extra subgroup.

By contrast, the three-group model balances these criteria effectively, yielding a BIC of −109777.53, an AIC of −109745.22, and an 8.9% smallest group proportion. All three trajectory groups demonstrate adequate AvePP values and maintain odds of correct classification above 5%. Moreover, each group exhibits a reasonably narrow confidence interval for its trajectory and maintains odds of correct classification above 5%. Taken together, these results suggest that the three-group solution offers the best combination of model fit, parsimony, and interpretability, capturing clinically meaningful differences in UCR trajectories without fragmenting the sample into extremely small or overlapping groups. We therefore selected the three-group model for our primary analyses.

Abbreviations: AIC, Akaike information criterion; AvePP, average posterior probability; BIC, Bayesian information criterion; GBTM, group-based trajectory model; UCR, urea to creatinine ratio

[Reference]

1. Jones BL, Nagin DS, Roeder K: **A SAS procedure based on mixture models for estimating developmental trajectories**. *Sociological methods & research* 2001, **29**(3):374-393.
2. Nagin DS, Nagin D: **Group-based modeling of development**: Harvard University Press; 2005.

Table S1. Diagnosis codes used in the study

Disease	ICD-9-CM codes and ICD-10-CM
Diabetes mellitus	250, 250.0, 250.1, 250.2, 250.3, 250.7, 250.00, 250.01, 250.10, 250.11, 250.20, 250.21, 250.30, 250.31, 250.70, 250.71, E10.1, E10.5, E10.6, E10.9, E11.0, E11.5, E11.6, E11.9
Hypertension	401, 401.0, 401.1, 401.9, 402, 402.0, 402.00, 402.01, 402.1, 402.10, 402.11, 402.9, 402.90, 402.91, 403, 403.0, 403.00, 403.01, 403.1, 403.10, 403.11, 403.9, 403.90, 403.91, 404, 404.0, 404.00, 404.01, 404.02, 404.03, 404.1, 404.10, 404.11, 404.12, 404.13, 404.9, 404.90, 404.91, 404.92, 404.93, 405, 405.0, 405.01, 405.09, 405.1, 405.11, 405.19, 405.9, 405.91, 405.99, I11.0, I11.9, I12.0, I12.9, I13.0, I13.11, I13.2, I15.0, I15.8, I15.1, N26.2, I15.2, I15.9, I10, I13.10
Acute respiratory distress syndrome	518.5, 518.81, 518.82, J95.1, J95.2, J95.3, J95.82, J96.0, J96.9, J80, Z87.09
Pleural effusion	511.1, 511.9, 511.0, J90, J94.8, J91.8, J92.0, J92.9, J94.1, J94.9, R09.1
Hypovolemic shock	785.59, R57.1
Periphral Vascular Disease	093.0, 437.3, 440, 441, 443.1, 443.2, 443.8, 443.9, 447.1, 557.1, 557.9, V43.4, I70, I71, I731, I738, I739, I771, I79.0, I79.2, K55.1, K55.8, K55.9, Z95.8, Z95.9
Congestive Heart Failure	398.91, 402.01, 402.11, 402.91, 404.01, 404.03, 404.11, 404.13, 404.91, 404.93, 425.4, 425.5, 425.7, 425.8, 425.9, 428, I43, I50, I09.9, I11.0, I13.0, I13.2, I25.5, I42.0, I42.5, I42.6, I42.7, I42.8, I42.9, P29.0
Cerebrovascular Disease	362.34, 430, 431, 432, 433, 434, 435, 436, 437, 438, G45, G46, I60, I61, I62, I63, I64, I65, I66, I67, I68, I69, H34.0
Sepsis (Infectious disease + Organ dysfunction)	Infectious disease<br>001, 002, 003, 004, 005, 008, 009, 010, 011, 012, 013, 014, 015, 016, 017, 018, 020, 021, 022, 023, 024, 025, 026, 027, 030, 031, 032, 033, 034, 035, 036, 037, 038, 039, 040, 041, 090, 091, 092, 093, 094, 095, 096, 097, 098, 100, 101, 102, 103, 104, 110, 111, 112, 114, 115, 116, 117, 118, 320, 322, 324, 325, 420, 421, 451, 461, 462, 463, 464, 465, 481, 482, 485, 486, 494, 510, 513, 540, 541, 542, 566, 567, 590, 597, 601, 614, 615, 616, 681, 682, 683, 686, 730, 5695, 5720, 5721, 5750, 5990, 7110, 7907, 9966, 9985, 9993, 49121, 56201, 56203, 56211, 56213, 56983, A000, A001, A009, A0100, A0101, A0102, A0103, A0104, A0105, A0109, A011, A012, A013, A014, A020, A021, A0220, A0221, A0222, A0223, A0224, A0225, A0229, A028, A029, A030, A031, A032, A033, A038, A039, A050, A051, A052, A058, A053, A055, A054, A059, A044, A040, A041, A042, A043, A048, A045, A046, A047, A049, A080, A082, A0811, A0819, A0831, A0832, A0839, A084, A088, A09, A157, A156, A150, A155, A154, A158, A159, A170, A171, A1781, A1782, A1783, A1789, A179, A1831, A1832, A1839, A1883, A1801, A1802, A1803, A1809, A1811, A1812, A1813, A1815, A1814, A1817, A1816, A1818, A1810, A184, A182, A1850, A1851, A1852, A1853, A1854, A1859, A186, A1881, A187, A1885, A1889, A1882, A1884, A190, A191, A192, A198, A199, A200, A201, A207, A202, A203, A208, A209, A210, A213, A212, A211, A217, A218, A219, A220, A221, A222, A227, A228, A229, A230, A231, A232, A233, A238,

	A239, A240, A241, A242, A243, A249, A250, A251, A259, A320, A3211, A3212, A327, A3281, A3282, A3289, A329, A260, A267, A268, A269, A280, A282, A288, A289, A305, A301, A300, A302, A303, A304, A308, A309, A310, A311, A312, A318, A319, A360, A361, A3689, A362, A3686, A3681, A3685, A363, A3682, A3683, A3684, A369, A3700, A3710, A3780, A3790, J020, J0300, J0301, A380, A381, A388, A389, A46, A390, A3981, A392, A393, A394, A391, A3950, A3953, A3951, A3952, A3982, A3983, A3984, A3989, A399, A35, A400, A401, A408, A409, R6510, R6511, R6520, A412, A4101, A4102, A411, A403, A414, A4150, A413, A4151, A4152, A4153, A4159, A4181, A4189, A419, A431, L081, A420, A430, A421, A422, B479, A4281, A4282, A4289, A438, A429, A439, B471, A480, A488, K9081, M60009, A482, A483, A484, A491, B955, B950, B951, B954, B952, B953, B958, A4901, A4902, B9561, B9562, B957, B961, B9620, B9621, B9622, B9623, B9629, A492, B963, A498, B964, B965, A493, B960, B966, B967, B9682, B9689, B9681, A499, A5001, A5002, A5003, A5004, A5005, A5006, A5007, A5008, A5009, A501, A502, A5031, A5040, A5043, A5045, A5042, A5041, A5049, A5030, A5032, A5039, A5044, A5051, A5052, A5053, A5054, A5055, A5056, A5057, A5059, A506, A507, A509, A510, A511, A512, A5131, A5139, A5149, A5143, A5146, A5145, A5141, A5132, A5142, A5144, A515, A519, A5201, A5202, A5203, A5206, A5204, A5205, A5209, A5200, A5211, A5217, A5213, A522, A5214, A5219, A5215, A5212, A5210, A523, A5271, A5272, A5274, A5275, A5277, A5278, A5273, A5276, A5279, A528, A529, A530, A539, A5400, A5402, A5409, A541, A5429, A5401, A5422, A5423, A5403, A5424, A5421, A5431, A5432, A5439, A5433, A5430, A5442, A5449, A5441, A5440, A5443, A545, A546, A5489, A5481, A5483, A5485, A5482, A5484, A5486, A549, A270, A2781, A2789, A279, A690, A691, A660, A661, A662, A663, A664, A665, A666, A667, A668, A669, A670, A671, A672, A673, A679, A65, A698, A699, B350, B351, B352, B356, B353, B354, B355, B358, B359, B360, B361, B362, B363, B368, B369, B370, B3783, B373, B3741, B3742, B3749, B372, B371, B377, B376, B3784, B375, B3781, B3782, B3789, B379, B380, B383, B3881, B384, B387, B3889, B381, B382, B389, B394, G02, H32, I32, I39, B390, B391, B392, B393, B395, J17, B399, B400, B401, B402, B403, B407, B4081, B4089, B409, B410, B417, B418, B419, B480, B481, B420, B421, B427, B4281, B4282, B4289, B429, B430, B431, B432, B438, B439, B441, B442, B447, B4489, B449, B484, B470, B450, B452, B453, B457, B458, B459, B482, B460, B461, B462, B463, B464, B465, B468, B469, B488, B483, B49, G000, G001, G002, G003, G01, G008, G009, G042, G030, G038, G031, G039, G060, G061, G062, G07, G08, I301, I309, I300, I308, I330, I339, I8000, I8001, I8002, I8003, I8010, I8011, I8012, I8013, I80201, I80202, I80203, I80209, I80221, I80222, I80223, I80229, I80231, I80232, I80233, I80239, I80291, I80292, I80293, I80299, I803, I80211, I80212, I80213, I80219, I808, I809, J0100, J0101, J0110, J0111, J0120, J0121, J0130, J0131, J0140, J0141, J0180, J0181, J0190, J0191, J028, J029, J0380, J0381, J0390, J0391, J040, J0430, J0431, J050, J0410, J0411, J042, J0510, J0511, J060, J069, J13, J181, J150, J151, J14, J154, J153, J1520, J15211, J1529, J158, J155, J156, A481, J159, J180, J188, J189, J440, J441, J479, J470, J471, J860, J869, J850, J851, J852, J853, K352, K353, K3580, K3589, K37, K36, K5700, K5712, K5701, K5713, K5720, K5732, K5740, K5752, K5792, K5721, K5733, K5781, K5793, K610, K611, K612, K613, K614, K67, K658, K650,

	K651, K652, K6811, K6819, K653, K654, K659, K689, K630, K631, K750, K751, K810, N110, N118, N10, N151, N2884, N2885, N2886, N119, N12, N136, N16, N159, N340, N341, N342, N343, N390, N410, N411, N412, N413, N51, N414, N418, N419, N7001, N7002, N7003, N7011, N7012, N7013, N7091, N7092, N7093, N730, N731, N732, N733, N736, N734, N738, N74, N735, N739, N710, N711, N719, N72, N760, N761, N762, N763, N94810, N771, N750, N758, N751, N764, N766, N770, N759, N765, N7681, N7689, L02511, L02512, L02519, L03011, L03012, L03019, L03021, L03022, L03029, L02611, L02612, L02619, L03031, L03032, L03039, L03041, L03042, L03049, K122, L0201, L03211, L03212, L0211, L03221, L03222, L02211, L02212, L02213, L02214, L02215, L02216, L02219, L03311, L03312, L03313, L03314, L03315, L03316, L03319, L03321, L03322, L03323, L03324, L03325, L03326, L03329, L02411, L02412, L02413, L02414, L02419, L03111, L03112, L03113, L03114, L03119, L03121, L03122, L03123, L03124, L03129, L0231, L03317, L03327, L02415, L02416, L03115, L03116, L03125, L03126, L02811, L02818, L03811, L03818, L03891, L03898, L0291, L0390, L0391, L983, L040, L041, L042, L043, L048, L049, L080, L88, L0881, L0889, L928, L980, B781, E832, L0882, L089, M0000, M0010, M0020, M0080, M009, M00011, M00012, M00019, M00111, M00112, M00119, M00211, M00212, M00219, M00811, M00812, M00819, M00021, M00022, M00029, M00121, M00122, M00129, M00221, M00222, M00229, M00821, M00822, M00829, M00031, M00032, M00039, M00131, M00132, M00139, M00231, M00232, M00239, M00831, M00832, M00839, M00041, M00042, M00049, M00141, M00142, M00149, M00241, M00242, M00249, M00841, M00842, M00849, M00051, M00052, M00059, M00151, M00152, M00159, M00251, M00252, M00259, M00851, M00852, M00859, M00061, M00062, M00069, M00161, M00162, M00169, M00261, M00262, M00269, M00861, M00862, M00869, M00071, M00072, M00079, M00171, M00172, M00179, M00271, M00272, M00279, M00871, M00872, M00879, M0008, M0018, M0028, M0088, M0009, M0019, M0029, M0089, M8600, M8610, M8620, M86011, M86012, M86019, M86111, M86112, M86119, M86211, M86212, M86219, M86021, M86022, M86029, M86121, M86122, M86129, M86221, M86222, M86229, M86031, M86032, M86039, M86131, M86132, M86139, M86231, M86232, M86239, M86041, M86042, M86049, M86141, M86142, M86149, M86241, M86242, M86249, M86051, M86052, M86059, M86151, M86152, M86159, M86251, M86252, M86259, M86061, M86062, M86069, M86161, M86162, M86169, M86261, M86262, M86269, M86071, M86072, M86079, M86171, M86172, M86179, M86271, M86272, M86279, M8608, M8618, M8628, M8609, M8619, M8629, M8630, M8640, M8650, M8660, M868X9, M86311, M86312, M86319, M86411, M86412, M86419, M86511, M86512, M86519, M86611, M86612, M86619, M868X1, M86321, M86322, M86329, M86421, M86422, M86429, M86521, M86522, M86529, M86621, M86622, M86629, M868X2, M86331, M86332, M86339, M86431, M86432, M86439, M86531, M86532, M86539, M86631, M86632, M86639, M868X3, M86341, M86342, M86349, M86441, M86442, M86449, M86541, M86542, M86549, M86641, M86642, M86649, M868X4, M86351, M86352, M86359, M86451, M86452, M86459, M86551, M86552, M86559, M86651, M86652, M86659, M868X5, M86361, M86362, M86369, M86461, M86462, M86469, M86561,

	M86562, M86569, M86661, M86662, M86669, M868X6, M86371, M86372, M86379, M86471, M86472, M86479, M86571, M86572, M86579, M86671, M86672, M86679, M868X7, M8638, M8648, M8658, M8668, M868X8, M8639, M8649, M8659, M8669, M868X0, M869, M4620, M4621, M4622, M4623, M4624, M4625, M4626, M4627, M4628, M8960, M89611, M89612, M89619, M89621, M89622, M89629, M89631, M89632, M89639, M89641, M89642, M89649, M89651, M89652, M89659, M89661, M89662, M89669, M89671, M89672, M89679, M8968, M8969, M9080, M90811, M90812, M90819, M90821, M90822, M90829, M90831, M90832, M90839, M90841, M90842, M90849, M90851, M90852, M90859, M90861, M90862, M90869, M90871, M90872, M90879, M9088, M9089, M4630, M4631, M4632, M4633, M4634, M4635, M4636, M4637, M4638, M4639, R7881, T8579XA, T826XXA, T827XXA, T80211A, T80212A, T80218A, T80219A, T8022XA, T8351XA, T8359XA, T836XXA, T8450XA, T8451XA, T8452XA, T8453XA, T8454XA, T8459XA, T8460XA, T84610A, T84611A, T84612A, T84613A, T84614A, T84615A, T84619A, T84620A, T84621A, T84622A, T84623A, T84624A, T84625A, T84629A, T8463XA, T8469XA, T847XXA, T8571XA, T8572XA, T86842, T814XXA, R5084, N980, T8029XA, T880XXA
	Organ dysfunction
	4580, 4588, 4589, 7855, 78551, 78559, 7963, 51881, 51882, 51885, 78609, 7991, 967, 9671, 9672, 9604, 9390, 580, 5800, 5804, 5808, 58081, 58089, 5809, 584, 5845, 5846, 5847, 5848, 5849, 586, 3995, 570, 5722, 5733, 5734, 293, 3481, 3483, 78001, 78009, 8914, 2862, 2866, 2869, 2873, 2874, 2875, 79092, 2762, I951, I9589, I959, R579, R570, H49811, H49812, H49813, R571, R578, R6521, R031, J9600, J9601, J9602, J9690, J9691, J9692, J80, R0600, R0609, R063, R0683, R0689, R092, T423X1A, T423X2A, T423X3A, T423X4A, T426X1A, T364X1A, T364X2A, T364X3A, T364X4A, T190XXA, T191XXA, N000, N001, N002, N003, N004, N005, N006, N007, N010, N011, N012, N013, N014, N015, N016, N017, N018, N019, N08, N008, N009, N170, N171, N172, N178, N179, N19, K7200, K7201, K761, K762, K7290, K7291, K710, K7110, K7111, K712, K713, K714, K7150, K7151, K716, K717, K718, K719, K752, K753, K7581, K7589, K759, K764, K763, F05, F062, F060, F0630, F0631, F0632, F0633, F0634, F064, F061, F53, F068, G931, G9340, G9341, G9349, I6783, E035, R4020', R402110, R402111, R402112, R402113, R402114, R402120, R402121, R402122, R402123, R402124, R402210, R402211, R402212, R402213, R402214, R402220, R402221, R402222, R402223, R402224, R402310, R402311, R402312, R402313, R402314, R402320, R402321, R402322, R402323, R402324, R402340, R402341, R402342, R402343, R402344, R400, R401, R410, D681, D65, D688, D689, D473, D693, D6941, D6942, D6949, D6951, D6959, D7582, D696, R791, E872
Appendicitis	540.0, 540.1, 540.9, 540.9, 541, 542, 543.0, 543.9, K35, K35.2, K35.3, K35.80, K35.89, K37, K36, K38.0, K38.1, K38.2, K38.3, K38.8, K38.9, E800-E844, V00-V99
	389, 389.01, 389.02, 389.03, 389.04, 389.08, 389.1, 389.11, 389.12, 389.14, 389.18, 389.2, 389.7, 389.8, 389.9, H90.0,
Traffic accident	H90.11, H90.12, H90.2, H90.5, H90.3, H90.41, H90.42, H90.6, H90.71, H90.72, H90.8, H91.3, H91.01, H91.0,
Deafness	H91.03, H91.09, H91.8X1, H91.8X2, H91.8X3, H91.8X9, H91.90, H91.91, H91.92, H91.93

Abbreviations: CAD, coronary artery disease; CKD, chronic kidney disease; CVA, cerebrovascular accident; HF, heart failure; ICD-9-CM, International Classification of Diseases, 9th Revision, Clinical Modification; ICD-10-CM, International Classification of Diseases, 10th Revision, Clinical Modification

Table S2. Medication codes used in this study

Medicine		ATC codes
Hypertension	ACEi	C09A, C09B
	ARB	C09C, C09D
	Alpha-adrenergic blocker	C02CA
	Beta blocking agents	C07
	CCB	C08
	Diuretics	C03
	Others	C02, C04
Anti-uric acid	Probenecid	M04AB01
	Benzbromarone	M04AB03
	Sulfinpyrazone	M04AB02
	Allopurinol	M04AA01
	Febuxostat	M04AA03
	Colchicine	M04AC01
Antiplatelet	Warfarin	B01AA03
	Heparin	B01AB01
	Dalteparin	B01AB04
	Albumin	B05AA01
	Plavix (Clopidogrel)	B01AC04
	Ticlopidine	B01AC05
Statin		C10AA

Abbreviations: ACEi, angiotensin converting enzyme inhibitor; ARB, angiotensin II receptor blocker; CCB, calcium channel blocker;

Table S3. Average posterior probabilities of group assignment and statistics of model fit. The trajectory models successfully met the fit diagnostic criteria, indicating their suitability for the data, with lower AIC and BIC. Strong alignment existed between each group's estimated probability and the proportion of study members assigned to it using the maximum posterior probability assignment rule. Confidence intervals for each group were reasonably narrow, and the odds of accurate classification, based on the posterior probabilities of group membership, indicated strong model accuracy.

	Group1	Group2	Group3	Group4	BIC	AIC	Lowest group%
2 groups	0.9886458	0.9334518	-	-	-110048.52	-110019.8	9.3%
3 groups	0.6893434	0.7878439	0.9380432	-	-109777.53	-109745.22	8.9%
4 groups	0.9383681	0.8435205	0.904191	0.9374321	-107095.64	-107023.84	1.0%

Abbreviation: AIC, Akaike Information Criterion; BIC, Bayesian Information Criterion

Table S4. Sensitivity analysis for risk of MAKEs among different trajectory groups

	Trajectory groups			
	Group 1*		Group 3*	
	HR (95% CI)	P	HR (95% CI)	P
Original model (n=9717)	1.30 (1.20 -1.41)	< 0.001	1.54 (1.38- 1.73)	< 0.001
Eligible subjects with PS for multiple treatments (n=9717)	1.13 (1.02-1.25)	0.019	1.23 (0.98-1.55)	0.070
Cox regression models with different covariates (n=9717)				
Model 1 (age and gender)	1.90 (1.77-2.05)	< 0.001	1.23 (1.11-1.37)	< 0.001
Model 2 (age, gender, and baseline kidney function)	1.39 (1.29-1.50)	< 0.001	1.68 (1.51-1.88)	< 0.001
Model 3 (age, gender, co-morbidities, and baseline kidney function)	1.31 (1.21-1.42)	< 0.001	1.67 (1.49-1.87)	< 0.001
Overlap weighting with different populations (n=8192)				
Model with 180-day landmark analysis	1.44 (1.30-1.59)	< 0.001	1.62 (1.40-1.88)	< 0.001

* Reference: Group 2

Abbreviations: CI, confidence interval; HR, hazard ratio; MAKE, major adverse kidney event; PS, propensity score

Table S5. Sensitivity analysis for risk of mortality among different trajectory groups

	Trajectory groups			
	Group 1*		Group 3*	
	HR (95% CI)	P	HR (95% CI)	P
Original model (n=9717)	0.84 (0.74 -0.95)	0.007	1.59 (1.41- 1.80)	< 0.001
Eligible subjects with PS for multiple treatments (n=9717)	0.84 (0.72-0.99)	0.034	1.71 (1.39-2.10)	< 0.001
Cox regression models with different covariates (n=9717)				
Model 1 (age and gender)	0.75 (0.67-0.85)	< 0.001	1.87 (1.67-2.09)	< 0.001
Model 2 (age, gender, and baseline kidney function)	0.78 (0.69-0.88)	< 0.001	1.95 (1.73-2.19)	< 0.001
Model 3 (age, gender, co-morbidities, and baseline kidney function)	0.77 (0.68-0.87)	< 0.001	1.83 (1.63-2.07)	< 0.001
Overlap weighting with different populations (n=8198)				
Model with 180-day landmark analysis	0.72 (0.58-0.89)	0.002	1.79 (1.51-2.13)	< 0.001

* Reference: Group 2

Abbreviations: CI, confidence interval; HR, hazard ratio; PS, propensity score

Table S6. Sensitivity analysis for risk of MACEs different trajectory groups

	Trajectory groups			
	Group 1*		Group 3*	
	HR (95% CI)	P	HR (95% CI)	P
Original model (n=9717)	0.93 (0.83-1.04)	0.194	1.57 (1.40-1.77)	< 0.001
Eligible subjects with PS for multiple treatments (n=9717)	0.90 (0.78-1.03)	0.120	1.64 (1.34-2.02)	< 0.001
Cox regression models with different covariates (n=9717)				
Model 1 (age and gender)	0.90 (0.82-0.99)	0.047	1.73 (1.55-1.93)	< 0.001
Model 2 (age, gender, and baseline kidney function)	0.87 (0.79-0.97)	0.012	1.89 (1.69-2.12)	< 0.001
Model 3 (age, gender, co-morbidities, and baseline kidney function)	0.90 (0.81-1.00)	0.057	1.75 (1.56-1.96)	< 0.001
Overlap weighting with different populations (n=8198)				
Model with 180-day landmark analysis	0.86 (0.73-1.03)	0.094	1.71 (1.45-2.01)	< 0.001

* Reference: Group 2

Abbreviations: CI, confidence interval; HR, hazard ratio; MACE, major adverse cardiac event; PS, propensity score

Table S7. Specificity analysis for risk of 4 independent events, lung cancer, pneumonia, traffic accident, and deafness among different trajectory groups

	Trajectory groups			
	Group 1*		Group 3*	
Incident events	HR (95% CI)	P	HR (95% CI)	P
Lung cancer (n=9717)	1.20 (0.54 – 2.65)	0.652	0.29 (0.04-2.20)	0.229
Pneumonia (n=9717)	0.98 (0.80-1.19)	0.817	1.13 (0.85-1.50)	0.416
Traffic accident (n=9717)	1.09 (0.81-1.47)	0.567	0.69 (0.41-1.15)	0.150
Deafness (n=9717)	1.20 (0.75-1.91)	0.450	0.67 (0.28-1.60)	0.370

* Reference: Group 2

Abbreviations: CI, confidence interval; HR, hazard ratio

Figure S1. UMAP visualization of three UCR trajectory groups

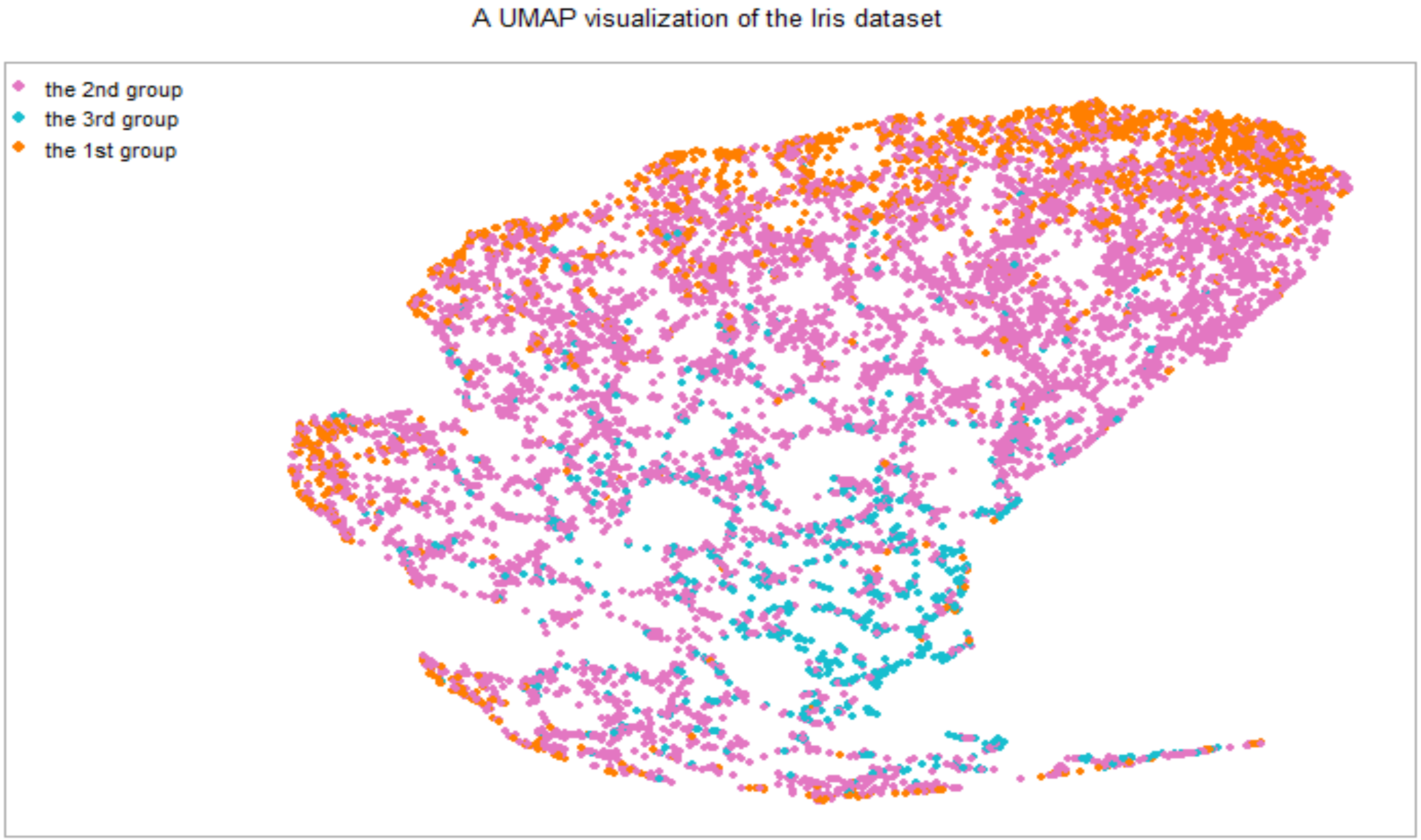

Abbreviations: UCR, urea to creatinine ratio; UMAP, Uniform manifold approximation and projection.

# Group 1 was low UCR, group 2 was middle and group 3 was high UCR.

Figure S2. Sankey diagrams showing the natural course of three UCR trajectory groups. (A) Baseline CKD – post-AKD UCR – clinical kidney outcome; (B) Baseline CKD – post-AKD UCR – clinical cardiovascular outcome

(A)

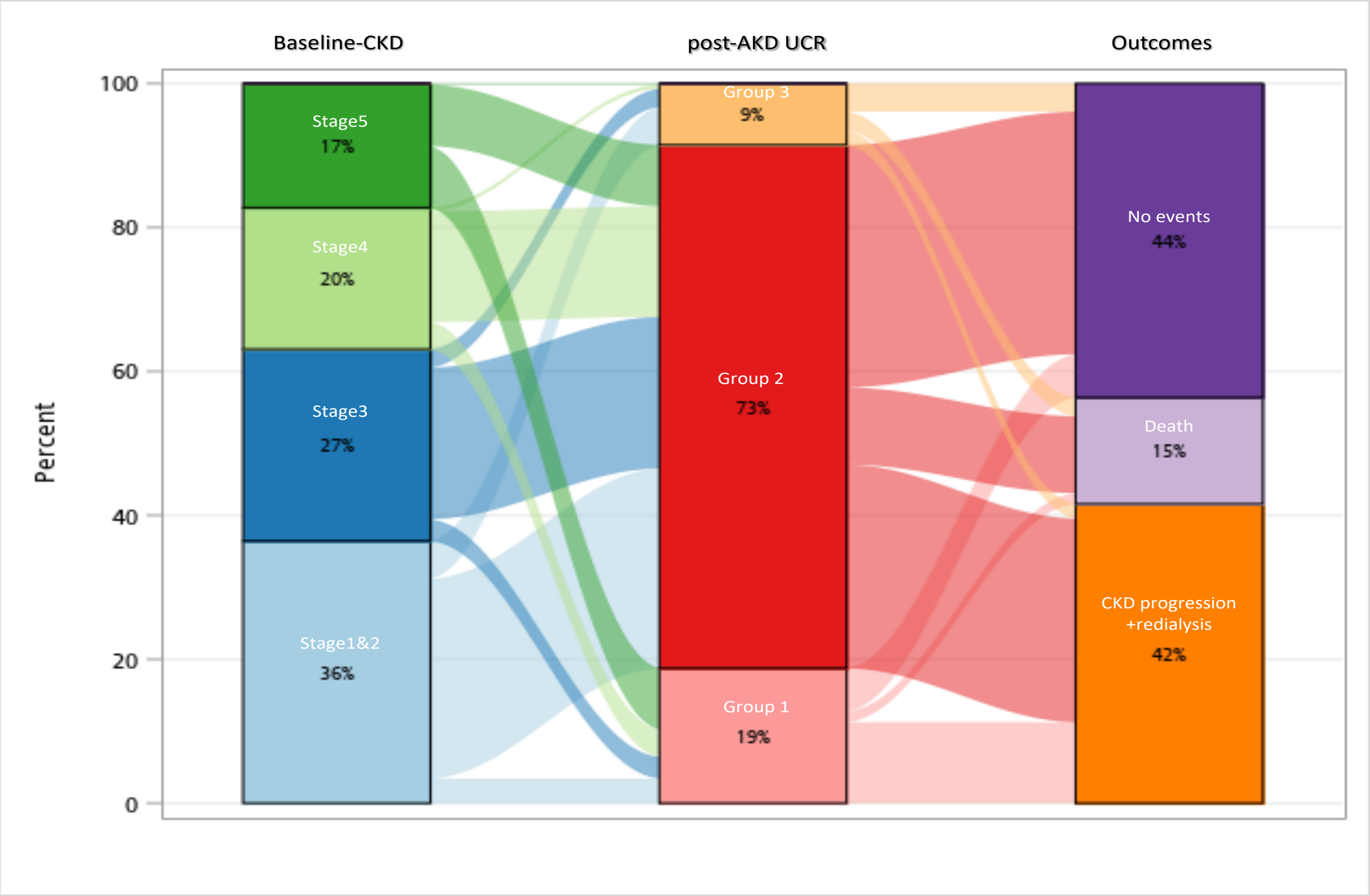

(B)

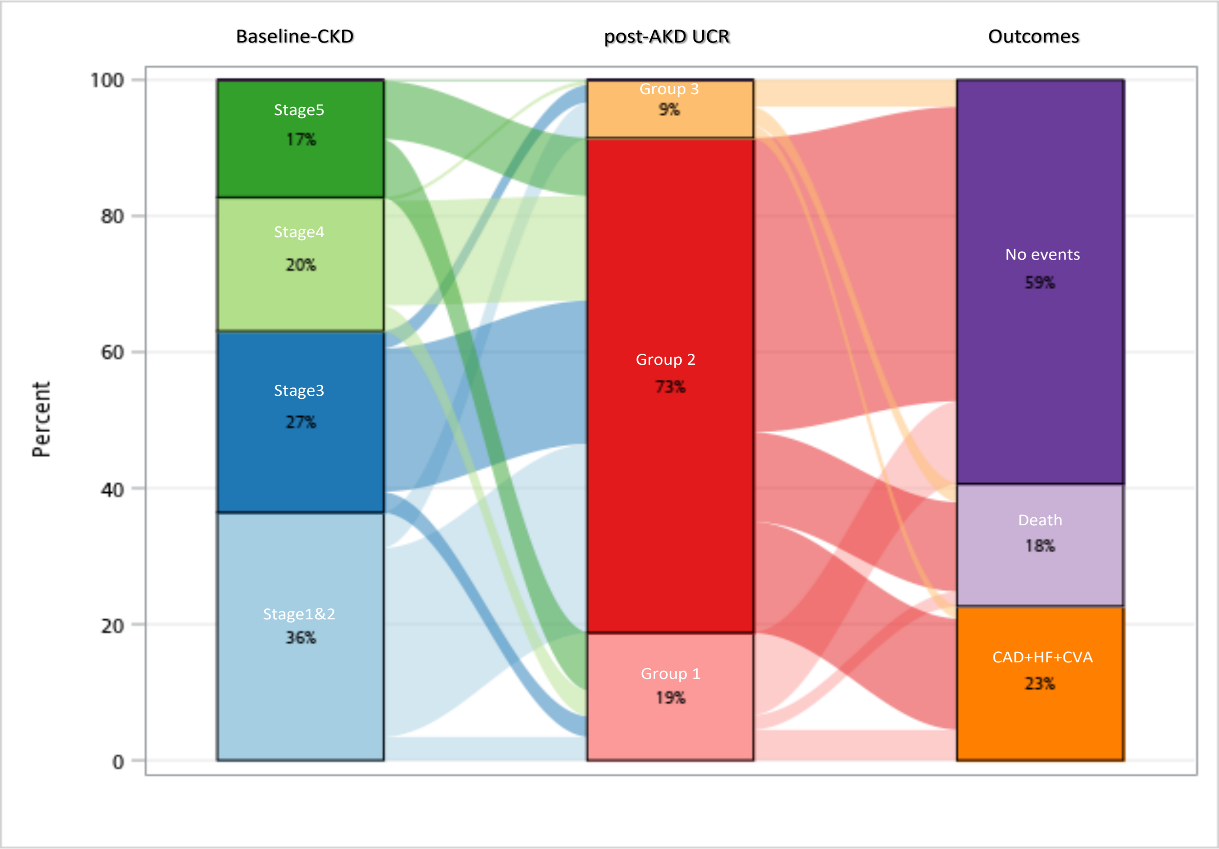

Abbreviations: AKD, acute kidney disease; CAD, coronary artery disease; CKD, chronic kidney disease; CVA, cerebrovascular accidents; HF, heart failure; UCR, urea to creatinine ratio.

Figure S3. Subgroup analysis of MAKEs

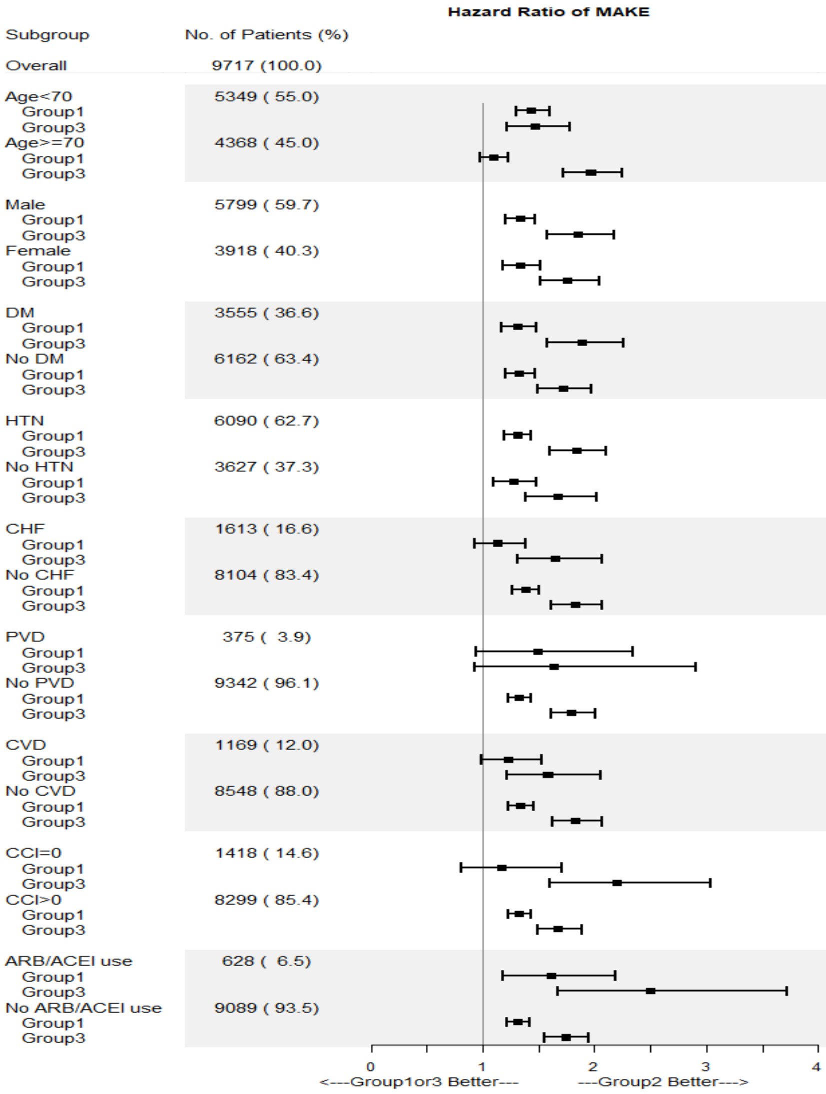

Subgroup	No. of Patients (%)	Cum. Event Rate Group2	Cum. Event Rate Group1 or 3	P Value
Overall	9717 (100.0)			
Age<70	5349 (55.0)			0.00
Group1		32.0	54.1	
Group3			35.2	
Age>=70	4368 (45.0)			
Group1		49.3	60.5	
Group3			56.2	
Male	5799 (59.7)			0.11
Group1		38.2	54.5	
Group3			48.0	
Female	3918 (40.3)			
Group1		42.6	59.9	
Group3			47.3	
DM	3555 (36.6)			0.09
Group1		46.1	63.1	
Group3			53.6	
No DM	6162 (63.4)			
Group1		36.4	52.2	
Group3			44.8	
HTN	6090 (62.7)			0.02
Group1		45.8	62.7	
Group3			51.5	
No HTN	3627 (37.3)			
Group1		30.6	43.2	
Group3			41.5	
CHF	1613 (16.6)			0.03
Group1		54.0	66.7	
Group3			56.2	
No CHF	8104 (83.4)			
Group1		36.9	55.0	
Group3			45.5	
PVD	375 (3.9)			0.21
Group1		35.9	56.9	
Group3			37.5	
No PVD	9342 (96.1)			
Group1		40.1	56.3	
Group3			48.1	
CVD	1169 (12.0)			0.16
Group1		47.6	63.0	
Group3			51.0	
No CVD	8548 (88.0)			
Group1		38.9	55.5	
Group3			46.9	
CCI=0	1418 (14.6)			0.00
Group1		16.2	18.0	
Group3			36.6	
CCI>0	8299 (85.4)			
Group1		44.3	60.6	
Group3			49.9	
ARB/ACEI use	628 (6.5)			0.68
Group1		48.1	63.1	
Group3			63.3	
No ARB/ACEI use	9089 (93.5)			
Group1		39.4	55.9	
Group3			46.6	

Abbreviations: ACEI, Angiotensin-converting enzyme inhibitors; ARB, angiotensin receptor blocker; CCI, Charlson Comorbidity Index; CHF, congestive heart failure; CVD, cardiovascular disease; DM, diabetes mellitus; HTN, hypertension; acute kidney disease; CAD, coronary artery disease; CKD, chronic kidney disease; CVA, cerebrovascular accidents; HF, heart failure; PVD, peripheral vascular disease; UCR, urea to creatinine ratio.

# Group 1 was low UCR, group 2 was middle and group 3 was high UCR.

Figure S4. Subgroup analysis of mortality

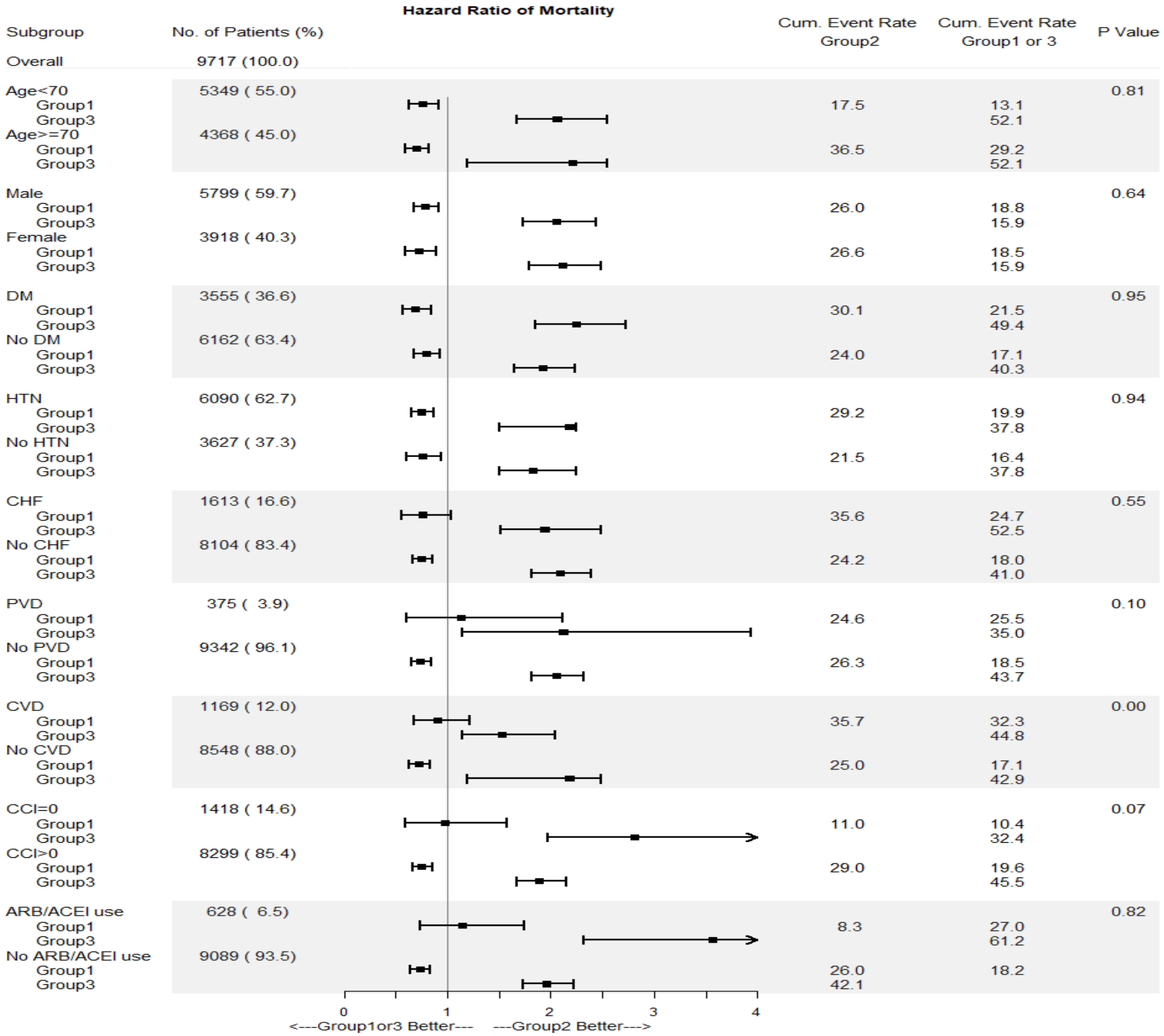

Abbreviations: ACEI, Angiotensin-converting enzyme inhibitors; ARB, angiotensin receptor blocker; CCI, Charlson Comorbidity Index; CHF, congestive heart failure; CVD, cardiovascular disease; DM, diabetes mellitus; HTN, hypertension; acute kidney disease; CAD, coronary artery disease; CKD, chronic kidney disease; CVA, cerebrovascular accidents; HF, heart failure; PVD, peripheral vascular disease; UCR, urea to creatinine ratio.

# Group 1 was low UCR, group 2 was middle and group 3 was high UCR.

Figure S5. Subgroup analysis of MACEs

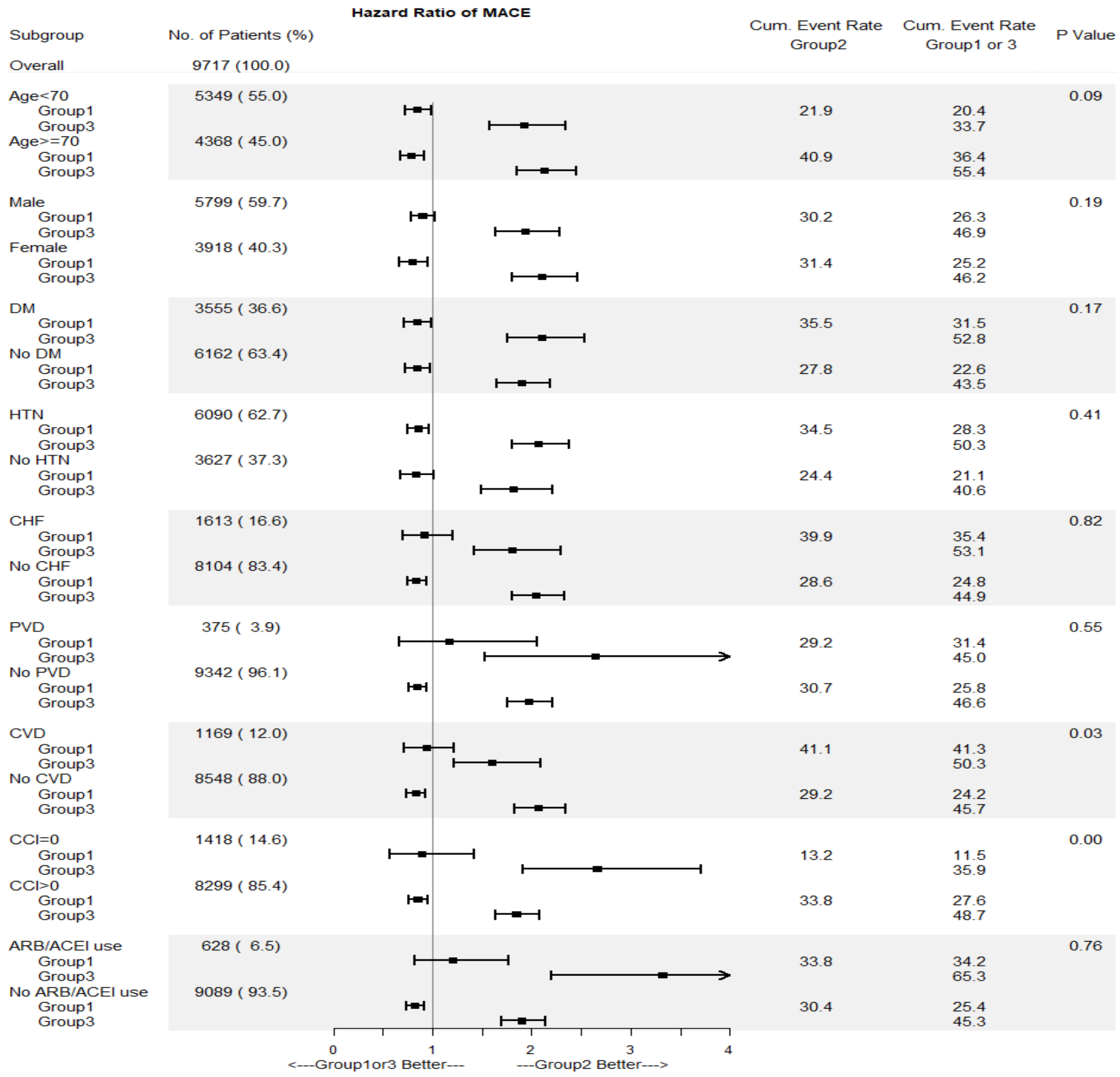

Abbreviations: ACEI, Angiotensin-converting enzyme inhibitors; ARB, angiotensin receptor blocker; CCI, Charlson Comorbidity Index; CHF, congestive heart failure; CVD, cardiovascular disease; DM, diabetes mellitus; HTN, hypertension; acute kidney disease; CAD, coronary artery disease; CKD, chronic kidney disease; CVA, cerebrovascular accidents; HF, heart failure; PVD, peripheral vascular disease; UCR, urea to creatinine ratio.

# Group 1 was low UCR, group 2 was middle and group 3 was high UCR.
